# Identifying phytopathogenic fungi in Al-Baha province, Saudi Arabia through their molecular and morphological features: An overview

**DOI:** 10.1016/j.sjbs.2023.103572

**Published:** 2023-01-26

**Authors:** Bandar Almiman

**Affiliations:** Department of Biology, Life science faculty, Al Baha University, Al Baha province, Saudi Arabia

**Keywords:** Al Baha Province, Plant Diversity, Mycotoxigenic species, Fungal plant pathogen, Optimal growth temperature, ITS rDNA Molecular identification and characterisation

## Abstract

Fungi are major pathogens of plants. They are responsible for most of the spoilage that occurs to plants in fields or in storage conditions. In addition to the direct impacts of fungi upon the plant’s fruiting body, such as leaf spot, wilt, rust, dieback and rot, fungi can contaminate plants with mycotoxins. Twenty isolates were molecularly identified in this study representing eight genera and twelve species. The most common species identified in this work belongs to *Aspergillus* (33.3%), *Penicillium* (16.6%) and *Fusarium* (16.6%) genera, which are well known to have mycotoxigenic species.

Environmental factors have a significant influence on the biological activity of fungi, including growth, sporulation and mycotoxin production. Temperature and water activity affect fungal virulence factors, such as growth, colonisation, spread and mycotoxin production. This work found the optimal temperature for the growth of isolates, was 30 °C for 75 % of isolates and at 25 °C for 25 % of isolates. This information is useful, as it helps to identify the phytopathogenic and mycotoxigenic species, and determining optimal growth temperatures is important to control them and reduce their threats.

## 1 Introduction

Due to its diverse geological topology and climate zone, the flora of the Kingdom of Saudi Arabia is rich and varied (Obaid et al., 2020). The number of plant species in Saudi Arabia is estimated to be around 2300 species that belong to 142 families (Collenette, 1999, Alzandi et al., 2021).

The Al Baha province, which is located in the southwest of Saudi Arabia, has six main cities: Al Baha, Alaqiq, Almandaq, Almikhwah, Baljurashi and Qilwah, of which Al Baha is the capital city of the Al Baha region (Alshehri, 2020). The ecological topology of this area is represented by a wide range of forests, mountains, valleys and wildlife areas. The vegetation of Al Baha province is estimated to be around 320 species, belonging to 228 genera and 75 families, representing wide range of fruits, vegetables, grains and medicinal plants (Al-Namazi et al., 2021). Agricultural practices are indigenous custom in Al Baha region for many decades, whether these are natural or cultivated plants (Collenette, 1999).

The impacts of global warming are a serious anxiety to humans, as it is perceived to affect animals and global yields of crops, by enhancing the virulence of pathogenic invasive microbes’ species (Bebber et al., 2013). Globally, plant pathogens are considered to be responsible for up to 40 % of lost crops annually, making them a significant factor in agriculture and food security (Takan et al., 2012, Bebber et al., 2013). The devastating effects of fungi upon plants ranks them as the second major plant pathogens; for example, fungi are responsible for about 50 % reduction in annually maize production (Fandohan et al., 2003). As well as directly reducing crop yields, fungi can also contaminate crops with mycotoxins. Mycotoxins are toxins produced by fungi, which are known to be harmful to the health of humans and animals. They have carcinogenic properties, as reported by the International Agency for Research (Nelson et al., 1993). According to the Food and Agriculture Organization (FAO), up to 50 % of the world’s annual crops are contaminated with mycotoxin (Fandohan et al., 2003). Dates are one of the most important crops grown in Saudi Arabia with approximately 28 million palm trees producing up to 14 % of the global date crop. However, post-harvest crop losses, due to fungi, range from 2.7 % to 33 % depending on the date varieties (El-Habbab et al., 2017). Moreover, a number of imported crops, such as bananas and mangos, stored in major Saudi Arabian depots were also infested with fungal pathogens and mycotoxins, becoming contaminated with toxicogenic species such as *Aspergillus niger, Aspergillus flavus, Colletotrichum musae* and *Penicillium spp* which led to further economic losses (Abd-Elsalam et al., 2010, Ahmed and Mohammed, 2014).

Environmental conditions can enhance or inhibit the biological activity of fungal pathogens. Thus, it is important to identify those conditions that are unfavourable to the pathogens, and use that knowledge to control them in the field, greenhouse and storage (Hawker, 1950, Chapin et al., 1987). The most influential environmental factors are temperature and water. These factors dramatically affect the biological activity of fungi, such as growth, sporulation and mycotoxin production, as well as affecting the plant’s immune system. There is considerable variation in the optimal temperature favoured by different fungi to enhance their sporulation, mycelium growth, invasion and colonisation of host tissues (Magan and Lacey, 1984, Gock et al., 2003).

The optimal temperatures are variable between different species and strains from the same species, thus identifying the optimal temperature will helps to build the right strategy to control them (Hawker, 1950, Chapin et al., 1987, Moore and Six, 2015). For example, two strains of *Fusarium culmorum* species isolated from maize from different geographical locations, showed 10° C variance in their optimal growth temperature (Hope et al., 2005).

**Fig. 1:**
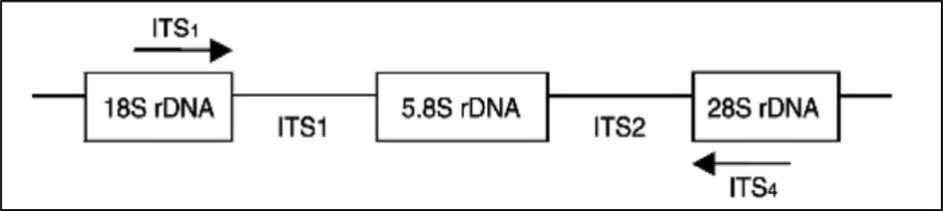
Illustrating the position of the internal transcribed spacer region (ITS), which is located between the small and the large ribosomal subunits (18S and 28S rRNA). The arrows indicate the coverage area for the forward primer, ITS1, and the reverse primer, ITS4 (Yan et al., 2011).

Identifying new isolate of fungal species through molecular mechanisms has greater accuracy than identifying them through their morphological characteristics (Taylor et al., 2000). The molecular species identification relies on bands variation exist on gel electrophoresis or relies on generating new sequence used for BLAST analysis to find similarity among species sequences that are available on gene banks, such as UNITE and National Centre for Biotechnology Information (NCBI) (Raja et al., 2017). The most useful universal marker for identifying fungal species is the internal transcribed spacer (ITS) marker, which is situated between the small and large subunits of ribosomal RNA (18S, 5.8S and 28S) (White et al., 1990). The ITS marker is popular due to its conserved region which exists in almost fungal genomes and its PCR fragment size which ranges from 400 to 900 bp (Arif et al., 2010, Cowan and Fay, 2012). Within ITS regions the most extensively favoured primers used for fungal molecular identification are the ITS1 and ITS4 primers due to their large coverage between the small subunit 18S and the large subunit 28S, which showed a high level of variation between different species (Fig. 1) (Yan et al., 2011). By 2012, the gene banks held more than 175,000 lengths of ITS sequences, representing over 15,000 fungal species (Schoch et al., 2012). However, by 2017, the available data for ITS sequences in gene banks still represented less than 1 % of the 5.1 million fungal species (Raja et al., 2017). In order to identify and define new fungal isolates and allocate them to the correct species classification using sequences generated from ITS markers, several scientists agree that the sequence homology should be between 96 % and 100 % (Mbareche et al., 2021). Recent work has focused on defining local fungal species based on their ITS markers and their phylogenetic evolutionary relationship with reference sequence isolates. In addition, identifying the optimal growth temperature of each isolate to contribute in building the right strategy to control them.

## 2 Materials and methods

### 2.1 Sample collection sites and sampling

The twenty fungal samples were collected from various sites related to crop production and storage within the Al-Bahah province, having previously obtained the owner's permission. Nineteen of the samples were collected from fruit, vegetables or crops in Al Bahah and Baljurashi, and one sample was collected from infected roses in Al Makhwah city. At each collection site a number of replicate samples were taken for the infested crop in order to confirm the causal agent responsible for the infestation. Therefore, the sampling procedure was designed to avoid mistakenly collecting transient isolates, e.g., spores attached to the soil or crop surface or transported on the wind.

**Fig. 2:**
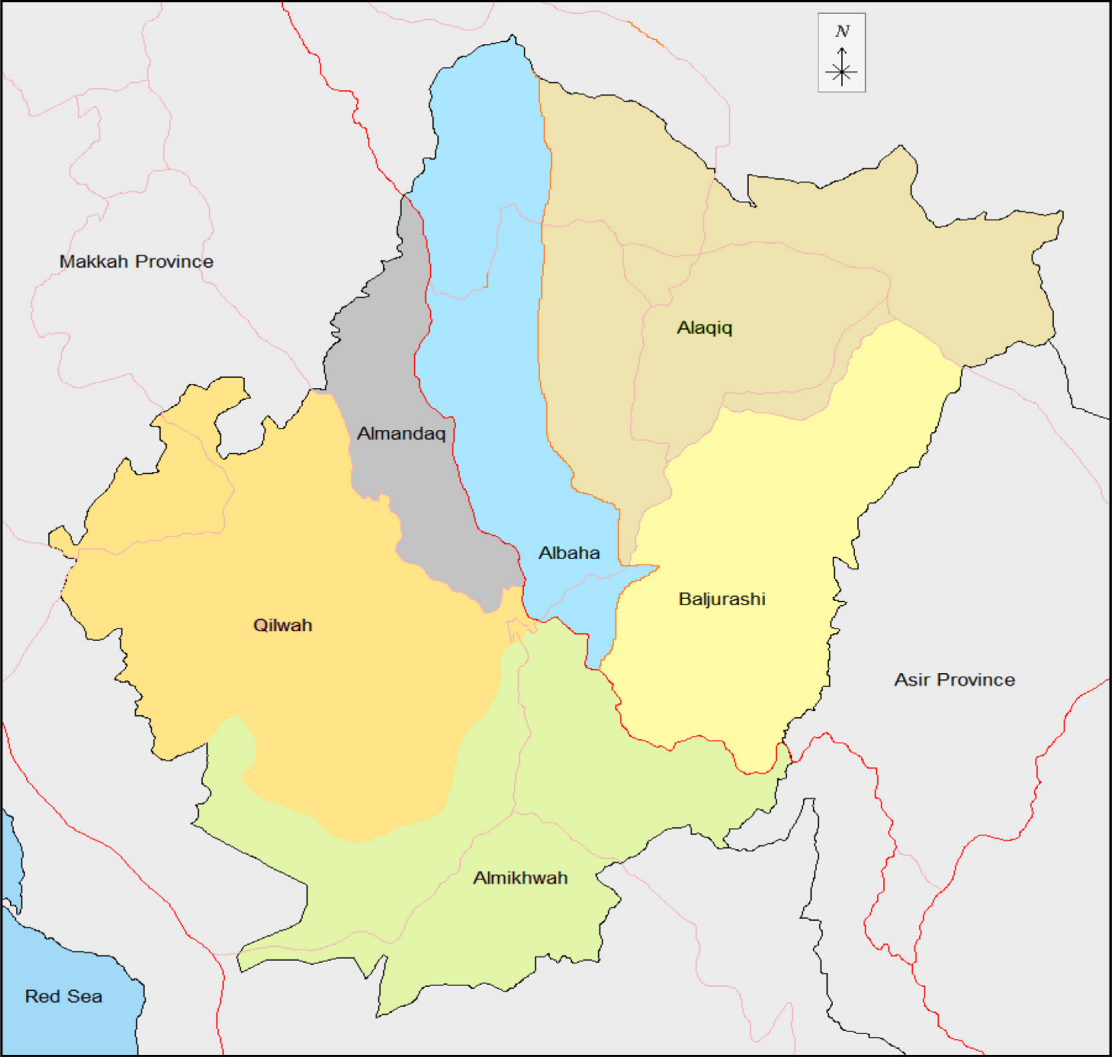
Map indicating the sampling sites for the 20 new fungal isolates collected from infected crops both in fields and in storage. The distance between Al Baha and Baljurashi being 60 km while Al Baha and Al Makhwah are 100 km apart.

The capital city of Al Baha province is Al Baha, it is located at longitude 41° 28′ 4E and latitude 20° 0′ 46 N in the elevation of 2,400 m above sea (Alshehri, 2020). The city of Baljurashi, it is located at longitude 41° 33′ 26E and latitude 19° 51′ 34 N in the elevation of 2,000 m above sea level. Whereas the location of the Almikhwah city at longitude 41° 26′ 8E and latitude 19° 46′ 46 N in about elevation of 447 m above sea level (Fig. 2) (Al-Namazi et al., 2021).

### 2.2 Isolation of fungi and stock culture preparation

The collected crops were initially washed with tap water, and then subsequently with 1 % sodium hypochlorite for two minutes to effectively disinfect the surface, before finally being thoroughly washed with distilled water. Once the crops were suitably prepared, a sterilized loop was used to scratch the surface of the infected tissue and to transfer spores or mycelium to a PDA medium for culturing. After five days of culturing on the PDA medium at 25 °C, inoculum of 0.5 mm from the margin was re-inoculated to a new PDA medium for subculturing in order to obtain a pure isolate culture (Abd-Elsalam et al., 2010, Sankar and Babu, 2012). Finally, stock cultures of new fungal isolates were created and stored in the Al-Baha University stock culture bank (Table 1).

**Table 1:** The information of new sequence isolated background along with Al Baha University culture collection code and NCBI accession.

	Fungal species	Origin	Host	Code	NCBI accession number
1	*Bipolaris prieskaensis*	Albaha	*Malus domestica*	BHU001	ON845652
2	*Fusarium oxysporum*	Albaha	*Prunus persica*	BHU007	ON845734
3	*Aspergillus niger*	Albaha	Citrullus colocynthis	BHU014	ON834317
4	*Aspergillus niger*	Albaha	*Prunus armeniaca*	BHU018	ON866737
5	*Aspergillus tubingensis*	Albaha	*Punica granatum*	BHU021	ON847317
6	*Aspergillus niger*	Albaha	*Rumex nervosus*	BHU022	ON866738
7	*Actinomucor elegans*	Albaha	*Citrus limon*	BHU030	ON834321
8	*Geotrichum candidum*	Albaha	*Punica granatum*	BHU074A	ON868706
9	*Aspergillus terreus*	Almikhwah	*Rosa damascene*	BHU033	ON868703
10	*Aspergillus terreus*	Baljurashi	*Vitis vinifera*	BHU006	ON845732
11	*Aspergillus niger*	Baljurashi	*Morus rubra*	BHU013	ON866891
12	*Aspergillus parvathecius*	Baljurashi	*Ocimum basilicum*	BHU016	ON847316
13	*Penicillium viridicatum*	Baljurashi	*Pyrus communis*	BHU027	ON845807
14	*Aspergillus terreus*	Baljurashi	*Prunus armeniaca*	BHU029	ON833481
15	*Alternaria alternata*	Baljurashi	*Solanum melongena*	BHU071	ON844330
16	*Geotrichum candidum*	Baljurashi	*Solanum lycopersicum*	BHU073	ON868765
17	*Alternaria alternata*	Baljurashi	*Capsicum annuum*	BHU115	ON844336
18	*Penicillium polonicum*	Baljurashi	*Citrus limon*	BHU116	ON844337
19	*Fusarium incarnatum*	Baljurashi	Cucumis sativus	BHU117	ON844338
20	*Hanseniaspora uvarum*	Baljurashi	*Prunus armeniaca*	BHU119	ON845446

### 2.3 DNA extraction

Prior to DNA extraction, the colonial mycelium was sub-cultured in potato dextrose broth (PDB) to get a larger yield of DNA. Subsequently, liquid nitrogen, and a pestle and mortar were used to grind the tissue into powder. Then 150–200 mg of ground tissue was added to a 2 ml Eppendorf tube for DNA extraction, the process of which followed the protocol provided by Thermo scientific GeneJET Plant Genomic DNA Purification Kit. Finally, the DNA was stored at −20 °C for future use (Saleh et al., 2017).

### 2.4 Molecular identification and phylogenetic tree

Both forward and reverse primer (ITS1: TCCGTAGGTGAACCTGCGG and ITS4: TCCTCCGCTTATTGATATGC) were used to set 20 and 50-µl PCR reactions under the following PCR conditions: 35x cycles, initial denaturation at 95 °C for 3 min, denaturation at 94 °C for 1 min, annealing at 60 °C for 1 min, extension at 72 °C for 1 min, final extension at 72 °C for 5 min and storage 16 °C indefinitely. The PCR components for the 50 µl-reaction were 2.5 µl DNA, 2.5 µl of 20 µM forward primer, 2.5 µl of 20 µM reverse primer, 5 µl of 10x reaction buffer, 1 µl dNTPs, 3 µl MgCl_2_, 0.25 µl Taq DNA polymerase and topped up to 50-µl with free DNAase and RNAase water. The same ingredients were for the 20 μl-reaction but at different amounts. For the negative control, the same PCR components and amounts were used, but DNA was replaced with the free DNAase and RNAase water. After completing the PCR cycles and prior to loading the samples onto gel, 5x of loading dye was added to the PCR products. Agarose gel was prepared by dissolving 1 mg of agarose powder in 100 ml of 1X TAE buffer in a microwave oven for 2 min. Then 5-µl of ethidium bromide (10 mg/ml) was added and the mixture was poured onto a gel tray to cool down and solidify. The gel tray was placed in a gel electrophoresis tank filled with 1X TAE buffer and plugged into a 90 V power for 60 min (Saleh et al., 2017, Yang et al., 2018).

**Fig. 3:**
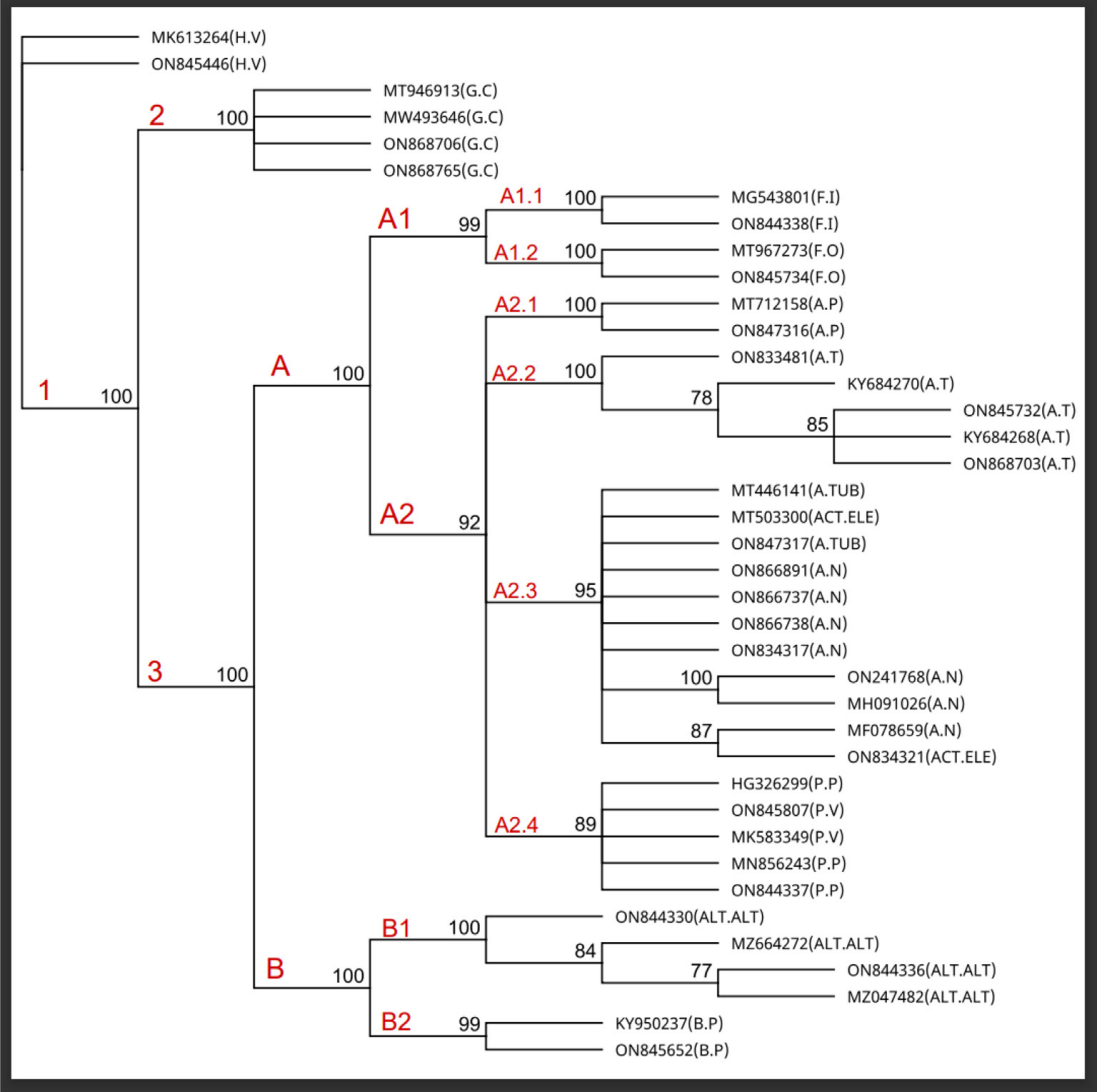
Neighbour-joining consensus tree based on the ITS genetic marker.

Subsequently, amplicons products were purified by the following the kite protocol (Qiagen QIAquick PCR Purification Kit). Then the PCR products were sent to an external sequencing service facility (Macrogen Inc.) for sequencing. The trace data were used to check the size and quality of these sequences and ambiguous bases were removed. Later the FASTA format were use in the BLAST analysis on NCBI or UNITE for species identification (Raja et al., 2017). Only sequences that showed above 96 % similarity on gene banks were downloaded and used as reference sequence for phylogenetic study (Table 2) (Mbareche et al., 2021). Both the original and reference sequences were used for multiple sequence alignments and to build a phylogenetic tree through Geneious prime package version 2022.2 (devolved by Biomatters) (Fig. 3). Using the MUSCLE alignment tool in Geneious prime, multiple sequences were aligned. The phylogenetic tree was constructed based on the distance tree and neighbour joining, using the Geneious tree builder algorithm. Genetic distance of tree by using Tamura-Nei model and Bootstrap supporting value set between 70 % and 100 % for consensus phylogenetic tree. The General Time-Reversible evolutionary model with 500 bootstrap replications was set for the best fit of consensus phylogenetic tree (Kearse et al., 2012, Al-Mutarrafi et al., 2019).

**Table 2:** The reference sequence isolates from NCBI.

	Fungal species	Origin	Host	Code	NCBI accession number
1	*Fusarium oxysporum*	Turkey Antakya	*Allium cepa*	OLAM3	MT967273
2	*Aspergillus niger*	Pakistan Rawalpindi	*Prunus persica*	ASN1	ON241768
3	*Bipolaris prieskaensis*	Iran Karaj	*Quercus*	32R2	KY950237
4	*Aspergillus terreus*	Oman Muscat	*Phaseolus radiatus*	SQU14026	KY684268
5	*Aspergillus niger*	Singapore Temasek	*Jatropha*	SG1	MH091026
6	*Aspergillus parvathecius*	Saudi Arabia Jeddah	*Afforestation*	Not provided	MT712158
7	*Aspergillus tubingensis*	China lanzhou	*Stipa purpurea*	ZMXR36	MT446141
8	*Aspergillus niger*	Turkey Kocaeli	*Lens culinaris*	MRC200804	MF078659
9	*Penicillium viridicatum*	Pakistan Punjab	*Malus domestica*	Not provided	MK583349
10	*Aspergillus terreus*	Oman Muscat	*Phaseolus vulgaris*	SQU14072	KY684270
11	*Actinomucor elegans*	China Gansu	*Avena sativa*	AS14	MT503300
12	*Alternaria alternata*	China hunan	*Japanese camellia*	YCB-8	MZ664272
13	*Geotrichum candidum*	China Anhui	*Actinidia deliciosa*	Gx3-1	MT946913
14	*Geotrichum candidum*	China Beijing	*Prunus persica*	BD01	MW493646
15	*Alternaria sp*	China Chengdu	*Ageratina Adenophora*	DCL02	MZ047482
16	*Penicillium verrucosum*	Pakistan Punjab	*Malus domestica*	Not provided	HG326299
17	*Penicillium polonicum*	China/ Nanjing	*Rubia cordifolia*	WZ-81	MN856243
18	*Fusarium incarnatum*	India GUJARAT	*Arachis hypogaea*	Jodhpur-2017–2	MG543801
19	*Hanseniaspora uvarum*	Turkey/Canakkale	*Punica granatum*	P-46	MK613264

All 20 sequences generated for this work have been deposited in NCBI gene bank and accession number for each isolate has been issued (Table 1). The uploading process for each sequence in NCBI has started after checking the sequence quality, quantity and reaching requirement criteria of submission and filling the proper information for each depositary sequence file separately.

### 2.5 Growth evaluation at three temperatures points

**Fig. 4:**
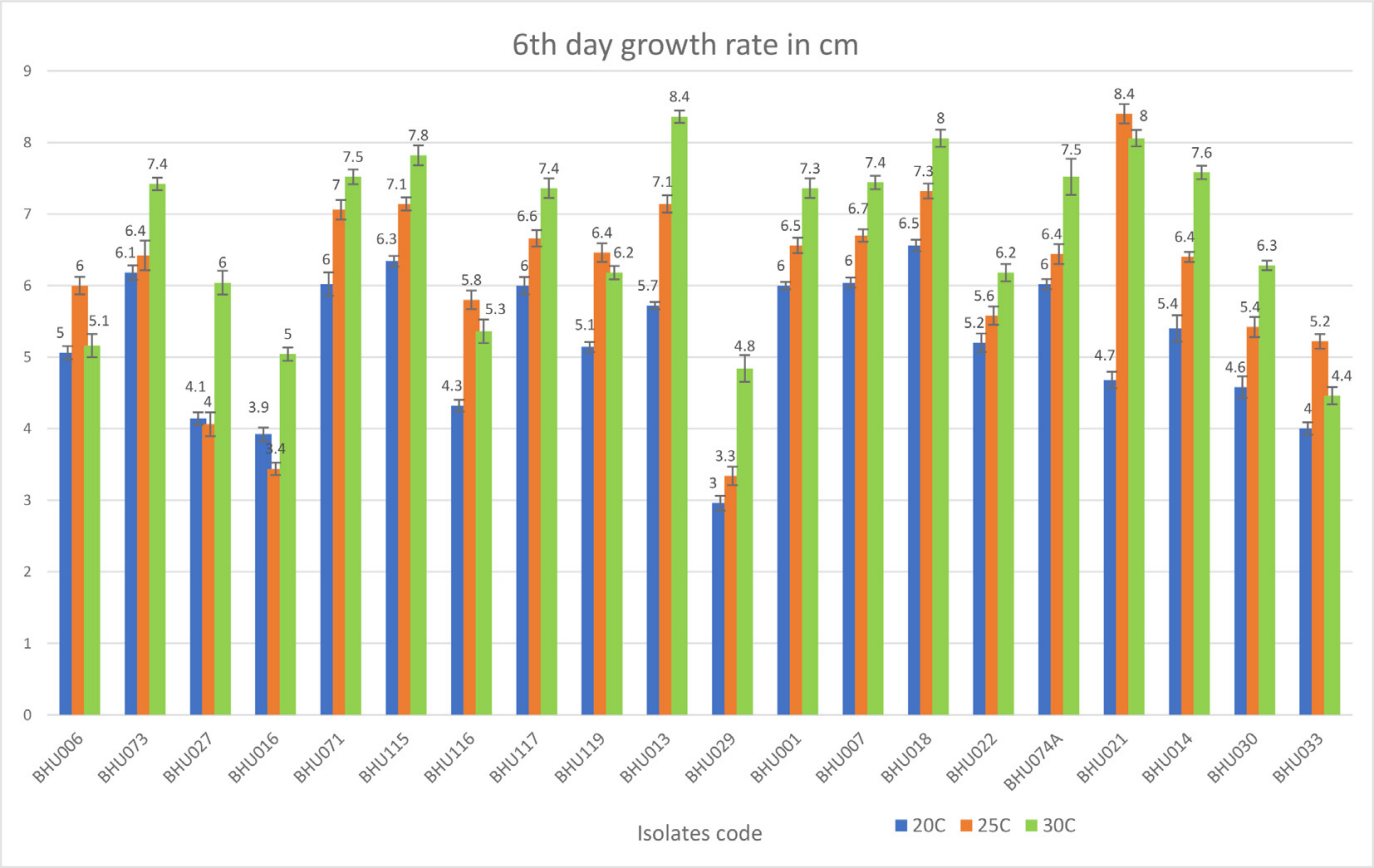
The effect different temperatures (20 °C, 25 °C and 30 °C) on the maximum growth rate for isolates originating from Albaha, Baljurashi and Almikhwah.

To establish the optimal growth temperature, the growth of the 20 isolates was assessed at different temperatures (20 °C, 25 °C and 30 °C). Isolates were cultured and sub-cultured on 9 cm PDA petri dishes. Later, 5 mm discs of inoculum from the margin were planted in the centre of new PDA petri dishes. For each isolate, there were five replicates for each of the three different temperatures. These were stored in an incubator for 6 days. At the end of the sixth day of incubation, radial growth measurements were taken for each replicate (Fig. 4). The growth results are shown in a bar chart, with the error bar represents the variation on replicates (Magan and Lacey, 1984, Gock et al., 2003, Moore and Six, 2015).

## 3 Results

### 3.1 Molecular identification and phylogenetic analysis using ITS region

The end-trimmed sequence length for 39 isolates, representing 12 species and eight genera based on the ITS genetic marker was ∼ 450 bp. The neighbour-joining consensus tree analysis distinguished the isolates of 12 species into three major nodes (1, 2 and 3) with 100 % bootstrap values (BSV) (Fig. 3). The first node represents a polyphyletic group with two taxa belong to *H. uvarum* species, while the second node represent monophyletic group of four taxa belong to *G. candidum* species. In contrast the third major node consist of two clades (A and B), which is subdivided into four main groups (A1, A2, B1 and B2) and eight subgroups (A1.1, A1.2, A2.1, A2.2, A2.3 and A2.4). The first clade A is the largest clade in terms of number of taxa (27 out of 39 isolates) distributed in two main groups (A1 and A2), with BSVs of between 92 % and 99 %. The group A1 was divided into two subgroups (A1.1 and A1.2), with BSVs of 99 %–100 %. The first subgroup, A1.1, has two taxa belonging to *F*. *incarnatum* and subgroup A1.2 has two taxa belonging to *F. oxysporum*. However, group A2 consist of four subgroups (A2.1, A2.2, A2.3 and A2.4) with BSVs of 78 %–100 %. The first subgroup, A2.1, was composed of two taxa belonging to *A. parvathecius* species, while the second subgroup, A2.2, was comprised of five taxa belonging to *A. terreus* species. In addition, the third subgroup, A2.3, represents a cluster of eleven taxa belonging to four species *A. terreus*, *A. tubingensis, A. niger* and *Actinomucor elegans.* Whereas the fourth subgroup, A2.4, was composed of five taxa, belonging to two closely related species, *P. polonicum* and *P. viridicatum*. Finally, clade B was composed only of two groups (B1 and B2), with BSVs between 77 % and100%, and without any subgroups. Group B1 consists of four taxa belonging to *Alternaria alternata* species; however, group B2 contains two taxa belonging to *B. prieskaensis* species.

### 3.2 Optimal growth temperature

The growth results for the 20 isolates reflect variability across the 12 species (12 group patterns) (Fig. 4). The growth rate of three isolates (BHU006, BHU029 and BHU033) belonging *to A. terreus,* are between 5 and 8.3 mm at 20 °C, 5.5–10 mm at 25 °C and 7.3–8.6 mm at 30 °C. Meanwhile, the two isolates belonging to *G. candidum* (BHU073 and BHU074A), recorded growth rates of 10–10.3 mm at 20 °C, 10.6–10.7 mm at 25 °C and 12.3–12.5 mm at 30 °C. On the other hand, one isolate (BHU027) belonging to *P. viridicatum,* showed growth of about 6.9 mm at 20 °C, 6.7 mm at 25 °C and 10 mm at 30 °C. In addition, there were two isolates (BHU115 and BHU071) belonging to *A. alternata*, which showed growth rates of 10–10.5 mm at 20 °C, 10.1–10.19 mm at 25 °C and 12.5–13 mm at 30 °C. Furthermore, one isolate (BHU116) belonging to *P. polonicum*, recorded a growth rate of 7.2 mm at 20 °C, 9.6 mm at 25 °C and 8.9 mm at 30 °C. Another single isolate (BHU117) belonging to *F. incarnatum* grew about 10 mm at 20 °C, 10.1 mm at 25 °C and 12.2 mm at 30 °C. Also, another single isolate (BHU119), belonging to *H. uvarum*, had a growth rate of 8.5 mm at 20 °C, 10.7 mm at 25 °C and 10.3 mm at 30 °C. Moreover, four isolates (BHU013, BHU018, BHU022 and BHU014) belonging to *A. niger* species had growth rates between 8.6 and10.1 mm at 20 °C, 9.3 – 12.2 mm at 25 °C and 10.3 – 13.9 mm at 30 °C. Whereas a single isolate (BHU001) belonging to *B. prieskaensis* grew by 10 mm at 20 °C, 10.9 mm at 25 °C and 12.2 mm at 30 °C. In addition, another single isolate (BHU007) belonging to *F. oxysporum*, recorded growth rates of 10 mm at 20 °C, 11.1 mm at 25 °C and 12.4 mm at 30 °C. Also, the growth rate of a single isolate (BHU021) belonging to *A. tubingensis* was 7.8 mm at 20 °C, 14 mm at 25 °C and 13.4 at 30 °C. Finally, another single isolate (BHU030) belonging to *A. elegans* species, showed a growth rate of 7.6 mm at 20 °C, 9 mm at 25 °C and 10.4 mm at 30 °C.

## 4 Discussion

### 4.1 Molecular identification using ITS rDNA region marker

The molecular identification of this work successfully defined the species of new isolates, with an accuracy rate of 97 %–99 % based on both NCBI and UNITE blast analysis. The topology of the phylogenetic tree (Fig. 3) describes the evolutionary relationship among the 39 isolates belong to 12 species and eight genera, and represent diverse hosts and geographical locations. The ITS marker has effectively distinguished between isolates at the species level, but it is less effective in distinguishing isolates within the species level. For example, isolates belong to *G. candidum*, *F*. *incarnatum*, *F*. *oxysporum*, *B. prieskaensis*, *P. polonicum* and *P. viridicatum* species were clustered together in groups, indicating reduced genetic variability between them. In contrast, isolates belonging to the same species, such as *A. alternata* showed genetic variabilities within species level. These results emphasis what is already mentioned in a number of publications about the efficiency of using ITS as a marker for interspecies-level comparisons, but it being less efficient at the intraspecies level. Most of the published works state the limitations of the ITS marker at the intraspecies level, such as *Fusarium* and *Penicillium* species (O'Donnell and Cigelnik, 1997, O'Donnell et al., 1998, Skouboe et al., 1999, O’Donnell et al., 2015). In contrast, few numbers of fungal species have showed effectiveness of ITS marker at both interspecies and intraspecies level such as *Alternaria spp* (Woudenberg et al., 2013, Armitage et al., 2015).

### 4.2 Optimal growth temperature

These growth results indicate that the optimum temperature for 15 out of 20 isolates was 30 °C, while the remaining five isolates (BHU006, BHU116, BHU019, BHU021 and BHU033) grew optimally at 25 °C. In terms of the growth rate and variability, it seems to be higher among different species and lower between the same species.

Most these of isolates find moderate to high temperatures to be favourable to growth, which is consistent with the dominant temperature at this geographical location. In contrast, the literature describing optimal growth conditions for isolates originating from Europe indicate that the preferred temperatures for growth are cold to moderate. For example, different *Fusarium* species isolates originated from variable geographical locations across Europe indicate the optimal growth temperature is between 15 °C and 25 °C (Hope et al., 2005, Samapundo et al., 2005). Whereas the 25 °C was the optimal temperature for growth for both *P. camemberti* and *P. roqueforti* species and *A. alternata* species (Balai and Ahir, 2013, Kalai et al., 2017). In contrast, several publications indicate that *Aspergillus* species, such as *A. flavus* and *A. niger* has a preference for temperatures between 30°Cand 35 °C (Alborch et al., 2011, Gasperini et al., 2022).

## 5 Conclusion and future perspectives

In conclusion, the study has highlighted *Aspergillus, Penicillium* and *Fusarium* spp as major candidates for infesting fruits and vegetables. These species are considered to be the most prolific mycotoxigenic species (Bennett and Klich, 2003). The optimal growth temperature for most isolates is identified as being between 25 °C and 30 °C. This information can help to build strategies to prevent their post- and pre-harvesting threats (Al-Mutarrafi et al., 2019).

In future research, the evaluation of mycotoxin production for species related to this work could prove productive. Future studies could employ common techniques such as high-performance liquid chromatography (HPLC) and ultra-performance liquid chromatography (UPLC) combined with suitable mycotoxin-inducing media to define whether or not these mycotoxins are acceptable for human consumption.

## Declaration of Competing Interest

The authors declare that they have no known competing financial interests or personal relationships that could have appeared to influence the work reported in this paper.
