# Supplementary material for: Identifying phytopathogenic fungi in Al-Baha province, Saudi Arabia through their molecular and morphological features: An overview
Source: Saudi J Biol Sci. 2023 Jan 26;30(3):103572. doi: 10.1016/j.sjbs.2023.103572 (PMC9898442; doi:10.1016/j.sjbs.2023.103572)
Supplement: Supplementary data 1 [file mmc1.pdf]

Sequence for multiple alignment 20 isolates + 19 reference sequences from NCBI and UNITE

>ON845652(B.P)

TGATCCGAGGTCAAAGTGAGAAATGGACTTGATGGA

TTGCTGACCTCTTTAGCTGGTTGTAAGCGCAAAAAATGTGCTGCGCTCCG

AAACCAGTAGGCCGGCTGCCAATCGTTTTAAGGCGAGTCTCGCGGCTAGG

CAAGACAAAAAAAACGCCCAACACCAGGCAAAGCTTGAGGGTACAAATGA

CGCTGGAACAGGCATGCCCTTTGGAATACCAAAGGGCGCAATGTGCGTTC

AAAGATTCGATGATTCACTGAATTCTGCAATTCACACTACGTATCGCATT

TCGCTGCGTTCTTCATCGATGCCGGAACCGAGAGATCCATTGTTGAAAGT

TGTAAATGATTTAACATTTGTTATACTGACGCTTATTGCAACTACTAAAA

AAAGGTTTATGGTGTGGTCCTGGTGGCGGGCGAACCCGCCCAGGAAACAA

CAAGTACTCATAACACTTGGGTGGAAAAATAGTTCACCCTCCGCCACTGT

>KY950237(B.P)

AGTATGCTTAAGTTCAGCGGGTATCCCTACCTGATCCGAGGTCAAAAGTGAGAAATGGACTTGATGGATTGCT
GACCTCTTTAGCTGGTTGTAAGCGCAAAAAATGTGCTGCGCTCCGAAACCAGTAGGCCGGCTGCCAATCGTTT
TAAGGCGAGTCTCGCGGCAAGGCAAGACAAAAAAAACGCCCAACACCAAGCAAAGCTTGAGGGTACAAATG
ACGCTCGAACAGGCATGCCCTTTGGAATACCAAAGGGCGCAATGTGCGTTCAAAGATTCGATGATTCACTGAA
TTCTGCAATTCACACTACGTATCGCATTTCGCTGCGTTCTTCATCGATGCCAGAACCAAGAGATCCGTTGTTGA
AAGTTGTAAATGATTTAACATTTGTTATACTGACGCTGATTGCAACTACATAAAAAAAGGTTTATGGTGTGGTC
CTGGTGGCGGGCGAACCCGCCCAGGAAACAACAAGTACGCAAAAGACATGGGTGGAAAAATAGTTCAGCCT
CCGCCACTGTGCCCGGAGGCGCCAGCGCATACAGCCTTCATATTTTTTGTGAA

> ON845732(A.T)

CAAGTTGCAAATAAATGCGTCGGCGGGCGCCGGCCGGGCCTACGGAGCGGAAGACGAAGCC

CCATACGCTCGAGGACCGGACGCGGTGCCGCCGCTGCCTTTCGGGCCCGT

CCCCCGGGAGCCGGGGGACGAGGGCCCAACACACAAGCCGGGCTTGAGGG

CAGCAATGACGCTCGGACAGGCATGCCCCCCGGAATACCAGGGGGCGCAA

TGTGCGTTCAAAGACTCGATGATTCACTGAATTCTGCAATTCACATTAGT

TATCGCATTTCGCTGCGTTCTTCATCGATGCCGGAACCAAGAGATCCATT

GTTGAAAGTTTTAACTGATTGCAAAGAATCACACTCAGACTGCAAGCTTT

CAGAACAGGGTTCATGTTGGGGTCTCCGGCGGGCACGGGCCCGGGGGCGA

GTCGCCCCCCGGCGGCCAGCAACGCTGGCGGGCCCGCCGAAGCAACAAGG

TACAATAGTCACGGGTGGGGGAGGGTTGGGCCATAAAGACCCGCACTCGG

TAATGATCCTTCCGCAACACCCCCCTAAAAGAGAGGTCAAT

>KY684268(A.T)

ATGATATGCTTAAGTTCAGCGGGTATCCCTACCTGATCCGAGGTCAACCTGGAAAAAAACAAGTTGCAAATAA
ATGCGTCGGCGGGCGCCGGCCGGGCCTACGGAGCGGAAGACGAAGCCCCATACGCTCGAGGACCGGACGC
GGTGCCGCCGCTGCCTTTCGGGCCCGTCCCCCGGGAGCCGGGGGACGAGGGCCCAACACACAAGCCGGGCT
TGAGGGCAGCAATGACGCTCGGACAGGCATGCCCCCCGGAATACCAGGGGGCGCAATGTGCGTTCAAAGAC
TCGATGATTCACTGAATTCTGCAATTCACATTAGTTATCGCATTTCGCTGCGTTCTTCATCGATGCCGGAACCAA
GAGATCCATTGTTGAAAGTTTTAACTGATTGCAAAGAATCACACTCAGACTGCAAGCTTTCAGAACAGGGTTC
ATGTTGGGGTCTCCGGCGGGCACGGGCCCGGGGGCGAGTCGCCCCCCGGCGGCCAGCAACGCTGGCGGGCC
CGCCGAAGCAACAAGGTACAATAGTCACGGGTGGGGAGGTTGGGCCATAAAGACCCGCACTCGGTAATGAT
CCTTCCGCA

>ON845734(F.O)

GATTCGAGGTCAACATTCAGAAGTTGGGTGTTT

TACGGCATGGCCGCGCCGCTTCCAGTTGCGAGGTGTTAGCTACTACGCAT

TGGAAGCTGCGGCGGGACCGCCCTGTATTTGAGGGACGGCGTGTGCCCAC

AGGGGGCTTCTGCCGATCCCCAACGCCGGCCCGGGGGCCTGAGGGTTGTA

ATGACGCTCGAACAGGCATGCCCGCCAGAATACTGGCGGGCGCAATGTGC

GTTCAAAGATTCGATGATTCACTGAATTCTGCAATTCACATTACTTATCG

CATTTCGCTGCGTTCTTCATCGATGCCTGAGCCTAGAGATCCGTTGTTGA

AAGTTTTAATTTATTTGCTTGTTTACTCAAAAAAACATTATAAAAACAGA

GTTAGGGGTCCTCTGGCGGGGGCGGCCCGTTGTTACAGGGCCGTCTGTTC

CCGCCGAAGCAACGTTTTAGGTATGTTCACAGGGTTGATGAGTTGTATAA

CTCGGTAATGATCCCTCC

>MT967273(F.O)

GCATTCTACCTGATTCGAGGTCAACATTCAGAAGTTGGGTGTTTTACGGCATGGCCGCGCCGCTCTCCAGTTG
CGAGGTGTTAGCTACTACGCAATGGAAGCTGCGGCGGGACCGCCACTGTATTTGAGGGACGGCGTGTGCCCA
CAGGGGGCTTCTGCCGATCCCCAACGCCAGGCCCGGGGGCCTGAGGGTTGTAATGACGCTCGAACAGGCATG
CCCGCCAGAATACTGGCGGGCGCAATGTGCGTTCAAAGATTCGATGATTCACTGAATTCTGCAATTCACATTA
CTTATCGCATTTCGCTGCGTTCTTCATCGATGCCAGAGCCAAGAGATCCGTTGTTGAAAGTTTTAATTTATTTGC
TTGTTTACTCAGAAAAACATTATAAAAACAGAGTTAGGGGTCCTCTGGCGGGGGCGGCCCGTTGTTACAGGG
CCGTCTGTTCCCGCCGAAGCAACGTTTTAGGTATGTTCACAGGGTTGATGAGTTGTATAACTCGGTAATGATCC
CTCCGCAGGCCCCCCCTACAGAAAAG

>ON866891(A.N)

AGGTCACCTGGAAGAATGGTTGGAAA

ACGTCGGCGGCGCCGGCCAATCCTACGAGCATGTGACAAAGCCCCATACG

CTCGAGGATCGGACGCGGTGCCGCCGCTGCCTTTCGGGCCCGTCCCCCCG

GAGAGGGGGACGGCGACCCAACACACAAGCCGGGCTTGAGGGCAGCAATG

ACGCTTGGACAGGCATGCCCCCCGGAATACCAGGGGGCGCAATGTGCGTT

CAAAGACTCGATGATTCACTGAATTCTGCAATTCACATTAGTTATCGCAT

TTCGCTGCGTTCTTCATCGATGCCGGAACCAAGAGATCCATTGTTGAAAG

TTTTAACTGATTGCATTCAATCAACTCAGACTGCACGCTTTCAGACAGTG

TTCGTGTTGGGGTCTCCGGCGGGCACGGGCCCGGGGGGCAAAGGCGCCCC

CCCGGCGGCCGACAAGCGGCGGGCCCGCCGAAGCAACAGGGTACAATAGA

>MH091026(A.N)

TTCCTCCGCTTATTGATATGCTTAAGTTCAGCGGGTATCCCTACCTGATCCGAGGTCACCTGGAAAGAATGGTT
GGAAAACGTCGGCAGGCGCCGGCCAATCCTACAGAGCATGTGACAAAGCCCCATACGCTCGAGGATCGGAC
GCGGTGCCGCCGCTGCCTTTCGGGCCCGTCCCCCCGGAGAGGGGGACGGCGACCCAACACACAAGCCGGGC
TTGAGGGCAGCAATGACGCTCGGACAGGCATGCCCCCCGGAATACCAGGGGGCGCAATGTGCGTTCAAAGA
CTCGATGATTCACTGAATTCTGCAATTCACATTAGTTATCGCATTTCGCTGCGTTCTTCATCGATGCCGGAACCA
AGAGATCCATTGTTGAAAGTTTTAACTGATTGCATTCAATCAACTCAGACTGCACGCTTTCAGACAGTGTTCGT
GTTGGGGTCTCCGGCGGGCACGGGCCCGGGGGGCAGAGGCGCCCCCCCGGCGGCCGACAAGCGGCGGGCC
CGCCGAAGCAACAGGGTACAATAGACACGGATGGGAGGTTGGGCCCAAAGGACCCGCACTCGGTAATGATC
CTTCCGCAGGTTCACCTACGGAAGGATCATTACNGAGTGCGGGTCCTTTGGGCCCAACCTCCCATCCGTGTCT
ATTGTACCCTGTTGCTTCGGCGGGCCCGCCGCTTGTCGGCCGCCGGGGGG

>ON834317(A.N)

AGGTCAACCTGGAAGAATGGTTGGAAAA

CGTCGGCAGGCGCCGGCCAATCCTACAGAGCATGTGACAAAGCCCCATAC

GCTCGAGGATCGGACGCGGTGCCGCCGCTGCCTTTCGGGCCCGTCCCCCC

GGAGAGGGGGACGGCGACCCAACACACAAGCCGGGCTTGAGGGCAGCAAT

GACGCTCGGACAGGCATGCCCCCCGGAATACCAGGGGGCGCAATGTGCGT

TCAAAGACTCGATGATTCACTGAATTCTGCAATTCACATTAGTTATCGCA

TTTCGCTGCGTTCTTCATCGATGCCGGAACCAAGAGATCCATTGTTGAAA

GTTTTAACTGATTGCATTCAATCAACTCAGACTGCACGCTTTCAGACAGT

GTTCGTGTTGGGGTCTCCGGCGGGCACGGGCCCGGGGGGCAGAGGCGCCC

CCCCGGCGGCCGACAAGCGGCGGGCCCGCCGAAGCAACAGGGTACAATAG

ACACGGATGGGGAGGGTTGGGCCCAAAGGACCCGCACTCGGTAATGATCC

TTCCGCA

>ON241768(A.N)

CCTACCTGATCCGAGGTCAACCTGGAAAGAATGGTTGGAAAACGTCGGCAGGCGCCGGCCAATCCTACAGAG
CATGTGACAAAGCCCCATACGCTCGAGGATCGGACGCGGTGCCGCCGCTGCCTTTCGGGCCCGTCCCCCCGG
AGAGGGGGACGGCGACCCAACACACAAGCCGGGCTTGAGGGCAGCAATGACGCTCGGACAGGCATGCCCCC
CGGAATACCAGGGGGCGCAATGTGCGTTCAAAGACTCGATGATTCACTGAATTCTGCAATTCACATTAGTTAT
CGCATTTCGCTGCGTTCTTCATCGATGCCGGAACCAAGAGATCCATTGTTGAAAGTTTTAACTGATTGCATTCA

ATCAACTCAGACTGCACGCTTTCAGACAGTGTTCGTGTTGGGGTCTCCGGCGGGCACGGGCCCGGGGGGCAG
AGGCGCCCCCCCGGCGGCCGACAAGCGGCGGGCCCGCCGAAGCAACAGGGTACAATAGACACGGATGGGA
GGTTGGCCCCAAAGGACCCGCACTCGGTAATGATCCTTCCGCAGGTTCACCTACGGAAGGATCATTACCGAGT
GCGGGTCCTTTGGGCCCAACCTCCCATCCGTGTCTATTGTACCCTGTTGCTTCGGCGGGCCCGCCGCTTGTCGG
CCGCCGGGGGGGCGCCTCTGCCCCCCGGGCCCGTGCCCGCCGGAGACCCCAACACGAACACTGTCTGAAAGC
GTGCAGTCTGAGTTGATTGAATGCAATCAGTTAAAACTTTCAACAATGGATCTCTTGGTTCCGGCATCGATGAA
GAACGCAGCGAAATGCGATAACTAATGTGAATTGCAGAATTCAGTGAATCATCGAGTCTTTGAACGCACATTG
CGCCCCCTGGTATTCCGGGGGGCATGCCTGTCCGAGCGTCATTGCTGCCCTCAAGCCCGGCTTGTGTGTTGGG
TCGCCGTCCCCCTCTCCGGGGGGACGGGCCCGAAAGGCAGCGGGGGCACCGGCGTCCGATCCTCGAGCGTTT
GGGGCTTTGTCACATGCTCCGTAGGATTGGCCGGCGCCTGCCGACGTTTTCCAACCATTCTTTCCAGGTTGACC

>ON847316(A.P)

ATCCGAGGTCACCTGAGAAAAATAAGGTTGG

AGACGCCGGCTGGCGCCCGGCCGGCCCTAATCGAGCGGGTGACAAAGCCC

CATACGCTCGAGGACCGGACACGGTGCCGCCGCTGCCTTTCGGGCCCGTC

CCCCGGGGGGGACGACGACCCAACACACAAGCCGGGCTTGAGGGCAGCAA

TGACGCTCGGACAGGCATGCCCCCCGGAATGCCAGGGGGCGCAATGTGCG

TTCAAAGACTCGATGATTCACTGAATTCTGCAATTCACATTACTTATCGC

AGTTCGCTGCGTTCTTCATCGATGCCGGAACCAAGAGATCCATTGTTGAA

AGTTTTGACTGATTTGTATTCAGGCTCAGACTGCATCACTCTCAGGCATG

AAGTTCAGTGG

>MT712158(A.P)

AGTTTGATCCATTCCTGATCCGAGGTCAACCTGAGAAAAATAAGGTTGGAGACGCCGGCTGGCGCCCGGCCG
GCCCTAATCGAGCGGGTGACAAAGCCCCATACGCTCGAGGACCGGACACGGTGCCGCCGCTGCCTTTCGGGC
CCGTCCCCCGGGGGGGACGACGACCCAACACACAAGCCGGGCTTGAGGGCAGCAATGACGCTCGGACAGGC
ATGCCCCCCGGAATGCCAGGGGGCGCAATGTGCGTTCAAAGACTCGATGATTCACTGAATTCTGCAATTCACA
TTACTTATCGCAGTTCGCTGCGTTCTTCATCGATGCCGGAACCAAGAGATCCATTGTTGAAAGTTTTGACTGAT
TTGTATTCAGGCTCAGACTGCATCACTCTCAGGCATGAAGTTCAGTGGTCCCCGGCGGCTCGCCCCTAGGGGG
CTCCCCGCCGAAGCAACAGTGTTAGGTAGTCACGGGTGGGAGGTTGGGCGCCCGGAGGCAGCCCGCACTCG
GCAATGATCCTTCCGCAGGTTCCCCTACGGAAGG

>ON866737(A.N)

ATCCGAGGTCAACCTGGAAAGAATGGTTG

GAAAACGTCGGCCGGCGCCGGCCAATCCTACAGAGCATGTGACAAAGCCC

CATACGCTCGAGGATCGGACGCGGTGCCGCCGCTGCCTTTCGGGCCCGTC

CCCCCGGAGAGGGGGACGGCGACCCAACACACAAGCCGGGCTTGAGGGCA

GCAATGACGCTCGGACAGGCATGCCCCCCGGAATACCAGGGGGCGCAATG

TGCGTTCAAAGACTCGATGATTCACTGAATTCTGCAATTCACATTAGTTA

TCGCATTTCGCTGCGTTCTTCATCGATGCCGGAACCAAGAGATCCATTGT

TGAAAGTTTTAACTGATTGCATTCAATCAACTCAGACTGCACGCTTTCAG

ACAGTGTTCGTGTTGGGGTCTCCGGCGGGCACGGGCCCGGGGGGCAGAGG

CGCCCCCCCGGCGGCCGACAAGCGGCGGGCCCGCCGAAGCAACAGGGTAC

AATAGACACGGATGGGAGGTTGGGCCCAAAGGACCCGCACTCGGTAATGA

TCCTTCCGCAGGTCAACCCTACAGAAGATCATTACCGAGTGCGGGTCCTT

TGGGCCCACCTCCCATCCTGTCTATGT

>ON847317(A.T)

GATCCGAGGTCAACCTGGAAAAAATGGTTGGAAA

ACGTCGGCGGCGCCGGCCAATCCTACAGAGCATGTGACAAAGCCCCATAC

GCTCGAGGATCGGACGCGGTGCCGCCGCTGCCTTTCGGGCCCGTCCCCCC

GGAGAGGGGGACGGCGACCCAACACACAAGCCGGGCTTGAGGGCAGCAAT

GACGCTCGGACAGGCATGCCCCCCGGAATACCAGGGGGCGCAATGTGCGT

TCAAAGACTCGATGATTCACTGAATTCTGCAATTCACATTAGTTATCGCA

TTTCGCTGCGTTCTTCATCGATGCCGGAACCAAGAGATCCATTGTTGAAA

GTTTTAACTGATTGCATTCAATCAACTCAGACTGCACGCTTTCAGACAGT

GTTCGTGTTGGGGTCTCCGGCGGGCACGGGCCCGGGGGGCAAAGGCGCCC

CCCCGGCGGCCGACAAGCGGCGGGCCCGCCGAAGCAACAGGGTATAATAG

ACACGGATGGGAGGTTGGGCCCAAAGGACCCGCACTCGGTAATGATCCTT

CCGCAGCACCCCCTCTCAGAGAAAGAA

>MT446141(A.T)

AGGATGGGCTCTACCTGATCCGAGGTCACCTGGAAAAAATGGTTGGAAAACGTCGGCAGGCGCCGGCCAATC
CTACAGAGCATGTGACAAAGCCCCATACGCTCGAGGATCGGACGCGGTGCCGCCGCTGCCTTTCGGGCCCGT
CCCCCCGGAGAGGGGGACGGCGACCCAACACACAAGCCGGGCTTGAGGGCAGCAATGACGCTCGGACAGGC
ATGCCCCCCGGAATACCAGGGGGCGCAATGTGCGTTCAAAGACTCGATGATTCACTGAATTCTGCAATTCACA
TTAGTTATCGCATTTCGCTGCGTTCTTCATCGATGCCGGAACCAAGAGATCCATTGTTGAAAGTTTTAACTGAT
TGCATTCAATCAACTCAGACTGCACGCTTTCAGACAGTGTTCGTGTTGGGGTCTCCGGCGGGCACGGGCCCGG
GGGGCAAAGGCGCCCCCCCGGCGGCCGACAAGCGGCGGGCCCGCCGAAGCAACAGGGTATAATAGACACG
GATGGGAGGTTGGGCCCAAAGGACCCGCACTCGGTAATGATCCTTCCGCAGGTTCACCTACGGAAACCTTGTT
ACGACTTTTACTTCCTCTAAATGGACCAAGA

>ON866738(A.N)

GATCCGAGGTCACCTGGAAAGAATGGTTGGAA

AACGTCGGCGGCGCCGGCCAATCCTACAGAGCATGTGACAAAGCCCCATA

CGCTCGAGGATCGGACGCGGTGCCGCCGCTGCCTTTCGGGCCCGTCCCCC

CGGAGAGGGGGACGGCGACCCAACACACAAGCCGGGCTTGAGGGCAGCAA

TGACGCTCGGACAGGCATGCCCCCCGGAATACCAGGGGGCGCAATGTGCG

TTCAAAGACTCGATGATTCACTGAATTCTGCAATTCACATTAGTTATCGC

ATTTCGCTGCGTTCTTCATCGATGCCGGAACCAAGAGATCCATTGTTGAA

AGTTTTAACTGATTGCATTCAATCAACTCAGACTGCACGCTTTCAGACAG

TGTTCGTGTTGGGGTCTCCGGCGGGCACGGGCCCGGGGGGCAGAGGCGCC

CCCCCGGCGGCCGACAAGCGGCGGGCCCGCCGAAGCAACAGGGTACAATA

GACACGGATGGGGGAGGTTGGGCCCAAAGGACCCGCACTCGGTAATGATC

CTTCCGCATCCCCCCCCCTACAGAGAGGATCATTACCGAGTGCGGGTCCT

TGGGCCCACCTCCCACCTGTCTATG

>MF078659(A.N)

GCGGGCACGGCCCGGGGGCAAGGCGCCCCCCGGCGGCCGACAGCGGCGGGCCCGCCGAAGCAACAGGGTA
TATAGACAGGATGGGAGGTTGGGCCCAAAGGACCCGCACTCGGTAATGATTCCTCCGGCCTATTGATATGCTT
AAGTTCAGCGGGTATCCCTACCTGATCCGAGGTCAACCTGGAAAAAATGGTTGGAAAACGTCGGCAGGCGCC
GGCCAATCCTACAGAGCATGTGACAAAGCCCCATACGCTCGAGGATCGGACGCGGTGCCGCCGCTGCCTTTC
GGGCCCGTCCCCCCGGAGAGGGGGACGGCGACCCAACACACAAGCCGGGCTTGAGGGCAGCAATGACGCTC
GGACAGGCATGCCCCCCGGAATACCAGGGGGCGCAATGTGCGTTCAAAGACTCGATGATTCACTGAATTCTG
CAATTCACATTAGTTATCGCATTTCGCTGCGTTCTTCATCGATGCCGGAACCAAGAGATCCATTGTTGAAAGTT
TTAACTGATTGCATTCAATCAACTCAGACTGCACGCTTTCAGACAGTGTTCGTGTTGGGGTCTCCGGCGGGCA
CGGGCCCGGGGGGCAAAGGCGCCCCCCCGGCGGCCGACAAGCGGCGGGCCCGCCGAAGCAACAGGGTATA
ATAGACACGGATGGGAGGTTGGGCCCAAAGGACCCGCACTCGGTAATGATCCTTCCGCAGGTCACCCTACGG
AAGCATTACCGAGTGCGGGTCCTTGGGCCCAACCTCCCATCCTGTCTATATACCCGTTGCTCGGCGGGCGCCC
TGTCGGCGCCGGGGGGCGCTTGCCCCGGGCCGTGCGCGA

>ON845807(P.V)

GATCCGAGGTCAACCTGGATAAAAATTTGGGTTGATC

GGCAAGCGCCGGCCGGGCCTACAGAGCGGGTGACAAAGCCCCATACGCTC

GAGGACCGGACGCGGTGCCGCCGCTGCCTTTCGGGCCCGTCCCCCGGAAT

CGGAGGACGGGGCCCAACACACAAGCCGTGCTTGAGGGCAGCAATGACGC

TCGGACAGGCATGCCCCCCGGAATACCAGGGGGCGCAATGTGCGTTCAAA

GACTCGATGATTCACTGAATTTGCAATTCACATTACGTATCGCATTTCGC

TGCGTTCTTCATCGATGCCGGAACCAAGAGATCCGTTGTTGAAAGTTTTA

AATAATTTATATTTTCACTCAGACTTCAATCTTCAGACAGAGTTCGAGGG

TGTCTTCGGCGGGCGCGGGCCCGGGGGCGTGAGCCCCCCGGCGGCCAGTT

AAGGCGGGCCCGCCGAAGCAACAAGGTAAAATAAACACGGGTGGGAGGTT

GGACCCAAAGGGCCCTCACTCGGTAATGATCCTTCCG

>MK583349(P.V)

GGGGCAATCCCTGTTGGTTTCTTTTCCTCCGCTTATTGATATGCTTAAGTTCAGCGGGTATCCCTACCTGATCCG
AGGTCAACCTGGATAAAAATTTGGGTTGATCGGCAAGCGCCGGCCGGGCCTACAGAGCGGGTGACAAAGCC
CCATACGCTCGAGGACCGGACGCGGTGCCGCCGCTGCCTTTCGGGCCCGTCCCCCGGAATCGGAGGACGGGG
CCCAACACACAAGCCGGGCTTGAGGGCAGCAATGACGCTCGGACAGGCATGCCCCCCGGAATACCAGGGGG
CGCAATGTGCGTTCAAAGACTCGATGATTCACTGAATTTGCAATTCACATTACGTATCGCATTTCGCTGCGTTC
TTCATCGATGCCGGAACCAAGAGATCCGTTGTTGAAAGTTTTAAATAATTTATATTTTCACTCAGACTTCAATCT
TCAGACAGAGTTCGAGGGTGTCTTCGGCGGGCGCGGGCCCGGGGGCGTGAGCCCCCCGGCGGCCAGTAAAG
GCGGGCCCGCCGAAGCAACAAGGTAAAATAAACACGGGTGGGAGGTTGGACCCAAAGGGCCCTCACTCGGT
AATGATCCTTCCGCAGGTTCACCTACGGA

>ON833481(A.T)

GTTGCAAATAAATGCGTCGGCGGGCGCCGGCCGGGCCTACGGAGCGGAAGACGAA

GCCCCATACGCTCGAGGACCGGACGCGGTGCCGCCGCTGCCTTTCGGGCC

CGTCCCCCGGGAGCCGGGGGACGAGGGCCCAACACACAAGCCGGGCTTGA

GGGCAGCAATGACGCTCGGACAGGCATGCCCCCCGGAATACCAGGGGGCG

CAATGTGCGTTCAAAGACTCGATGATTCACTGAATTCTGCAATTCACATT

AGTTATCGCATTTCGCTGCGTTCTTCATCGATGCCGGAACCAAGAGATCC

ATTGTTGAAAGTTTTAACTGATTGCAAAGAATCACACTCAGACTGCAAGC

TTTCAGAACAGGGTTCATGTTGGGGTCTCCGGCGGGCACGGGCCCGGGGG

CGAGTCGCCCCCCGGCGGCCAGCAACGCTGGCGGGCCCGCCGAAGCAACA

AGGTACAATAGTCACGGGTGGGAGGTTGGGCCATAAAGACCCGCACTCGG

TAATGATCCTTCCGCAGGT

>KY684270(A.T)

TATTGATATGCTTAAGTTCAGCGGGTATCCCTACCTGATCCGAGGTCAACCTGGAAAAAAACAAGTTGCAAAT
AAATGCGTCGGCGGGCGCCGGCCGGGCCTACGGAGCGGAAGACGAAGCCCCATACGCTCGAGGACCGGAC
GCGGTGCCGCCGCTGCCTTTCGGGCCCGTCCCCCGGGAGCCGGGGGACGAGGGCCCAACACACAAGCCGGG
CTTGAGGGCAGCAATGACGCTCGGACAGGCATGCCCCCCGGAATACCAGGGGGCGCAATGTGCGTTCAAAG
ACTCGATGATTCACTGAATTCTGCAATTCACATTAGTTATCGCATTTCGCTGCGTTCTTCATCGATGCCGGAACC
AAGAGATCCATTGTTGAAAGTTTTAACTGATTGCAAAGAATCACACTCAGACTGCAAGCTTTCAGAACAGGGT
TCATGTTGGGGTCTCCGGCGGGCACGGGCCCGGGGGCGAGTCGCCCCCCGGCGGCCAGCAACGCTGGCGGG
CCCGCCGAAGCAACAAGGTACAATAGTCACGGGTGGGAGGTTGGGCCATAAAGACCCGCACTCGGTAATGAT
CCTTCCGCAGGTCACCCTACGG

>ON834321(ACT.ELE)

ATCCGAGGTCACCTGGAAAAATGGTTGGAAAACG

TCGGCAGGCGCCGGCCAATCCTACAGAGCATGTGACAAAGCCCCATACGC

TCGAGGATCGGACGCGGTGCCGCCGCTGCCTTTCGGGCCCGTCCCCCCGG

AGAGGGGGACGGCGACCCAACACACAAGCCGGGCTTGAGGGCAGCAATGA

CGCTCGGACAGGCATGCCCCCCGGAATACCAGGGGGCGCAATGTGCGTTC

AAAGACTCGATGATTCACTGAATTCTGCAATTCACATTAGTTATCGCATT

TCGCTGCGTTCTTCATCGATGCCGGAACCAAGAGATCCATTGTTGAAAGT

TTTAACTGATTGCATTCAATCAACTCAGACTGCACGCTTTCAGACAGTGT

TCGTGTTGGGGTCTCCGGCGGGCACGGGCCCGGGGGGCAAAGGCGCCCCC

CCGGCGGCCGACAAGCGGCGGGCCCGCCGAAGCAACAGGGTATAATAGAC

ACGGATGGGGAGGTTGGGCCCAAAGGACCCGCACTCGGTAATGATCCTTC

CGCAGC

>MT503300(ACT.ELE)

TTCCTCCCCGCTTTTTTGATATGCTTAAGTTCAGCGGGTATCCCTACCTGATCCGAGGTCAACCTGGAAAAAAT
GGTTGGAAAACGTCGGCAGGCGCCGGCCAATCCTACAGAGCATGTGACAAAGCCCCATACGCTCGAGGATCG
GACGCGGTGCCGCCGCTGCCTTTCGGGCCCGTCCCCCCGGAGAGGGGGACGGCGACCCAACACACAAGCCG
GGCTTGAGGGCAGCAATGACGCTCGGACAGGCATGCCCCCCGGAATACCAGGGGGCGCAATGTGCGTTCAA
AGACTCGATGATTCACTGAATTCTGCAATTCACATTAGTTATCGCATTTCGCTGCGTTCTTCATCGATGCCGGA
ACCAAGAGATCCATTGTTGAAAGTTTTAACTGATTGCATTCAATCAACTCAGACTGCACGCTTTCAGACAGTGT
TCGTGTTGGGGTCTCCGGCGGGCACGGGCCCGGGGGGCAAAGGCGCCCCCCCGGCGGCCGACAAGCGGCG
GGCCCGCCGAAGCAACAGGGTATAATAGACACGGATGGGAGGTTGGGCCCAAAGGACCCGCACTCGGTAAT
GATCCTTCCGCAGGCACCCCCCTAAACGAAAG

>ON868703(A.T)

ACAAGTTGCAAATAAATGCGTCGGCGGGCGCCGGCCGGGCCTACGGAGCGGAAGACGAAGC

CCCATACGCTCGAGGACCGGACGCGGTGCCGCCGCTGCCTTTCGGGCCCG

TCCCCCGGGAGCCGGGGGACGAGGGCCCAACACACAAGCCGGGCTTGAGG

GCAGCAATGACGCTCGGACAGGCATGCCCCCCGGAATACCAGGGGGCGCA

ATGTGCGTTCAAAGACTCGATGATTCACTGAATTCTGCAATTCACATTAG

TTATCGCATTTCGCTGCGTTCTTCATCGATGCCGGAACCAAGAGATCCAT

TGTTGAAAGTTTTAACTGATTGCAAAGAATCACACTCAGACTGCAAGCTT

TCAGAACAGGGTTCATGTTGGGGTCTCCGGCGGGCACGGGCCCGGGGGCG

AGTCGCCCCCCGGCGGCCAGCAACGCTGGCGGGCCCGCCGAAGCAACAAG

GTACAATAGTCACGGGTGGGGAGGTTGGGCCATAAAGACCCGCACTCGGT

AATGATCCTTCCGCAG

>ON844330(ALT.ALT)

CTGATCGAGGTCAAAGTTGAAAAGGCTTAATGGATGCTA

GACCTTTGCTGATAGAGAGTGCGACTTGTGCTGCGCTCCGAAACCAGTAG

GCCGGCTGCCAATTACTTTAAGGCGAGTCTCCAGCAAAGCTAGAGACAAG

ACGCCCAACACCAAGCAAAGCTTGAGGGTACAAATGACGCTCGAACAGGC

ATGCCCTTTGGAATACCAAAGGGCGCAATGTGCGTTCAAAGATTCGATGA

TTCACTGAATTCTGCAATTCACACTACTTATCGCATTTCGCTGCGTTCTT

CATCGATGCCAGAACCAAGAGATCCGTTGTTGAAAGTTGTAATTATTAAT

TTGTTACTGACGCTGATTGCAATTACAAAAGGTTTATGTTTGTCCTAGTG

GTGGGCGAACCCACCAAGGAAACAAGAAGTACGCAAAAGACAAGGGTGAA

TAATTCAGCAAGGCTGTAACCCCGAGAGGTTCCAGCCCGCCTTCATATTT

GTGTAATGATCCCTCCGCAT

>MZ664272(ALT.ALT)

GGGGGGATCTACTGATCCGAGGTCAAAGTTGAAAAAAAGGCTTAATGGATGCTAGACCTTTGCTGATAGAGA
GTGCGACTTGTGCTGCGCTCCGAAACCAGTAGGCCGGCTGCCAATTACTTTAAGGCGAGTCTCCAGCAAAGCT
AGAGACAAGACGCCCAACACCAAGCAAAGCTTGAGGGTACAAATGACGCTCGAACAGGCATGCCCTTTGGAA
TACCAAAGGGCGCAATGTGCGTTCAAAGATTCGATGATTCACTGAATTCTGCAATTCACACTACTTATCGCATT
TCGCTGCGTTCTTCATCGATGCCAGAACCAAGAGATCCGTTGTTGAAAGTTGTAATTATTAATTTGTTACTGAC
GCTGATTGCAATTACAAAAGGTTTATGTTTGTCCTAGTGGTGGGCGAACCCACCAAGGAAACAAGAAGTACG
CAAAAGACAAGGGTGAATAATTCAGCAAGGCTGTAACCCCGAGAGGTTCCAGCCCGCCTTCATATTTGTGTAA
TGATCCCTCCGCAGGTCCCCCTACGGAAG

>ON868765(G.C)

GATCTGAGGTTGAATAGTGTTGTTTTTCAAACG

AATTTGATTCGATTTTTTAGAAAAGCAATGCAATTCCAAGAGAGAAACAA

CGCTCAAACAAGTATACTTTGGGGGATACCCCAAAGTGCAATGTGCGTTC

AAAAACTGATGATTCACTTCTGCAATTCACAAGAAATATCGCGTTTCGCT

GCGTTCTTCATCGATACGAGAACCAAGAGATCCATTGTTAAAAGTTTTGA

TTATTTTTGTTTTGATTGTATGATTATTGTTTGCTGTGGTAATTTCACAA

ATATTTTCAATTCTTAATGATCCTTCCGCAGGTCACCCTACGGAAAAGGA

TCATTAAGAATTGAAAATATTTGTGAAATTACCACAGCAAACAATAATCA

TACTATCAAAACAAAAATAATCAAAACTTTTAACAATGGATCTCTTGGTT

CTCGTATCGA

>MT946913(G.C)

AGCTGCCCTTCCTCCGCTTATTGATATGCTTAAGTTCAGCGGGTAATCCTACTTGATCTGAGGTTGAATAGTGT
TGTTTTTCAAACGAATTTGATTCGAAATTTTAGAAAAGCAATGCAATTCCAAGAGAGAAACAACGCTCAAACA
AGTATACTTTGGGGGATACCCCAAAGTGCAATGTGCGTTCAAAAACTGATGATTCACTTCTGCAATTCACAAG
AAATATCGCGTTTCGCTGCGTTCTTCATCGATACGAGAACCAAGAGATCCATTGTTAAAAGTTTTGATTATTTTT
GTTTTGATTGTATGATTATTGTTTGCTGTGGTAATTTCACAAATATTTATAATTCTTAATGATCCTTCCGCAGGTT
CACCTACGGAAAGGGCAGCT

>ON868706(G.C)

TGATCTGAGGTTGATAGTGTTGTTTTTCAAACGAA

TTTGATTCGATTTTTTAGAAAAGCAATGCAATTCCAAGAGAGAAACAACG

CTCAAACAAGTATACTTTGGGGGATACCCCAAAGTGCAATGTGCGTTCAA

AAACTGATGATTCACTTCTGCAATTCACAAGAAATATCGCGTTTCGCTGC

GTTCTTCATCGATACGAGAACCAAGAGATCCATTGTTAAAAGTTTTGATT

ATTTTTGTTTTGATTGTATGATTATTGTTTGCTGTGGTAATTTCACAAAT

ATTTTCAATTCTTAATGATCCTTCCGCAGGT

>MW493646(G.C)

TCCTCCGCTTATTGATATGCTTAAGTTCAGCGGGTAATCCTACTTGATCTGAGGTTGAATAGTGTTGTTTTTCAA
ACGAATTTGATTCGATTTTTTAGAAAAGCAATGCAATTCCAAGAGAGAAACAACGCTCAAACAAGTATACTTT
GGGGGATACCCCAAAGTGCAATGTGCGTTCAAAAACTGATGATTCACTTCTGCAATTCACAAGAAATATCGCG
TTTCGCTGCGTTCTTCATCGATACGAGAACCAAGAGATCCATTGTTAAAAGTTTTGATTATTTTTGTTTTGATTG
TATGATTATTGTTTGCTGTGTAAATTTCACAAATATTATCAATTCTTAATGATCCTTCCGCAGGTTCACCTACGG
AAACCTTGTTACGACTTTTACTTCC

>ON844336(ALT.ALT)

TACTGATCCGAGGTCAAAGTTGAAAAAGAGGCTTAATGGAT

GCTAGACCTTTGCTGATAGAGAGTGCGACTTGTGCTGCGCTCCGAAACCA

GTAGGCCGGCTGCCAATTACTTTAAGGCGAGTCTCCAGCAAAGCTAGAGA

CAAGACGCCCAACACCAAGCAAAGCTTGAGGGTACAAATGACGCTTGAAC

AGGCATGCCCTTTGGAATACCAAAGGGCGCAATGTGCGTTCAAAGATTCG

ATGATTCACTGAATTCTGCAATTCACACTACTTATCGCATTTCGCTGCGT

TCTTCATCGATGCCAGAACCAAGAGATCCGTTGTTGAAAGTTGTAATTAT

TAATTTGTTACTGACGCTAATTGCAATTACAAAAGGTTTATGTTTGTCCT

AGTGGTGGGCGAACCCACCAAGGAAACAAGAAGTACGCAAAAGACAAGGG

TGAATAATTCAGCAAGGCTGTAACCCCGAGAGGTTCCAGCCCGCCTTCAT

ATTTGTGTAATGATCCCTCCG

>MZ047482(ALT.ALT)

AGGGGATCTACTGATCCGAGGTCAAAGTTGAAAAAAAGGCTTAATGGATGCTAGACCTTTGCTGATAGAGAG
TGCGACTTGTGCTGCGCTCCGAAACCAGTAGGCCGGCTGCCAATTACTTTAAGGCGAGTCTCCAGCAAAGCTA
GAGACAAGACGCCCAACACCAAGCAAAGCTTGAGGGTACAAATGACGCTCGAACAGGCATGCCCTTTGGAAT
ACCAAAGGGCGCAATGTGCGTTCAAAGATTCGATGATTCACTGAATTCTGCAATTCACACTACTTATCGCATTT
CGCTGCGTTCTTCATCGATGCCAGAACCAAGAGATCCGTTGTTGAAAGTTGTAATTATTAATTTGTTACTGACG
CTGATTGCAATTACAAAAGGTTTATGTTTGTCCTAGTGGTGGGCGAACCCACCAAGGAAACAAGAAGTACGCA
AAAGACAAGGGTGAATAATTCAGCAAGGCTGTAACCCCGAGAGGTTCCAGCCCGCCTTCATATTTGTGTAATG
ATCCCTCCGCAGGTTCACCTACGGAGACCTTGTTACGATTTTTAACTTCCAAA

>ON844337(P.P)

TACCTGATCCGAGGTCACCTGGATAAAAATTTGGGTTGA

TCGGCAAGCGCCGGCCGGGCCTACAGAGCGGGTGACAAAGCCCCATACGC

TCGAGGACCGGACGCGGTGCCGCCGCTGCCTTTCGGGCCCGTCCCCCGGA

ATCGGAGGACGGGGCCCAACACACAAGCCGGGCTTGAGGGCAGCAATGAC

GCTCGGACAGGCATGCCCCCCGGAATACCAGGGGGCGCAATGTGCGTTCA

AAGACTCGATGATTCACTGAATTTGCAATTCACATTACGTATCGCATTTC

GCTGCGTTCTTCATCGATGCCGGAACCAAGAGATCCGTTGTTGAAAGTTT

TAAATAATTTATATTTTCACTCAGACTTCAATCTTCAGACAGAGTTCGGG

GGTGTCTTCGGCGGGCGCGGGCCCGGGGGCGTGAGCCCCCCGGCGGCCAG

TAAAGGCGGGCCCGCCGAAGCAACAAGGTAAAATAAACACGGGTGGGGGA

GGTTGGACCCAAAGGGCCCTCACTCGGTAATGATCCTTCC

>MN856243(P.P)

TTCCTCCCCCTTTTGATATGCTTAAGTTCAGCGGGTATCCCTACCTGATCCGAGGTCAACCTGGATAAAAATTT
GGGTTGATCGGCAAGCGCCGGCCGGGCCTACAGAGCGGGTGACAAAGCCCCATACGCTCGAGGACCGGACG
CGGTGCCGCCGCTGCCTTTCGGGCCCGTCCCCCGGAATCGGAGGACGGGGCCCAACACACAAGCCGGGCTTG
AGGGCAGCAATGACGCTCGGACAGGCATGCCCCCCGGAATACCAGGGGGCGCAATGTGCGTTCAAAGACTC
GATGATTCACTGAATTTGCAATTCACATTACGTATCGCATTTCGCTGCGTTCTTCATCGATGCCGGAACCAAGA
GATCCGTTGTTGAAAGTTTTAAATAATTTATATTTTCACTCAGACTTCAATCTTCAGACAGAGTTCGGGGGTGT
CTTCGGCGGGCGCGGGCCCGGGGGCGTGAGCCCCCCGGCGGCCAGTAAAGGCGGGCCCGCCGAAGCAACA
AGGTAAAATAAACACGGGTGGGAGGTTGGACCCAAAGGGCCCTCACTCGGTAATGATCCTTCCGCAGGCACC
CCTAACGGAAG

>HG326299(P.P)

TTTTTTTTTTTCTTTGCCTTCCCCCCGTTGGTATTATATAGAGGGTGTGCTCCCCCCCCCCTTTGGGGGGAAACA
CCGAAAAAAATTTTGTGTGCGTGCGCCGCGCCGGGGGGGGCTAAAAAGAGAGAGGACAAAACCCCCCCTCC
CGGGGGAGGGACGGGGGTTCGGTGTCTCTTTTGGGCCTCCCCCCCGGAGATAGGAGAGGGGCCCCCCCCAC
ACCACCCGCTGGGCCCAAAAGTCTTGGACAGCCTTGGCCCCCCGGCATGCCCGGGGCCATTGGTTTAAAAGGT
CGGATATTTCACGGAATTGCCAATTTCACCTTTAGGTATTCCATTTGCGTGTTTTTTTAATGCAGCGCAACCCCA
AGGTTTCCTTGTTGGGAGGTTTTTAAATTTTTTTTTTTTTTCCTTCCGGGCTTCCTTCTTCGGGCCGGTTGGGGG
TTTTTTGGCGGGGCGGGCCGGGGGCTGGGCCCCCGGGCGGCCGGTAAGGCGGCCCGGCGGGGCCCGGGTT
ACCGGGTGGGGGGTGGCCCCCGGGGGGCCTCTGGTACCTTTCGGGTAGGGGTGCTGCGGAAGGATCATTAC
CGAGTGAGGGCCCTTTGGGTCCAACCTCCCACCCGTGTTTATTTTACCTTGTTGCTTCGGCGGGCCCGCCTTTA
CTGGCCGCCGGGGGGCTCACGCCCCCGGGCCCGCGCCCGCCGAAGACACCCCCGAACTCTGTCTGAAGATTG
AAGTCTGAGTGAAAATATAAATTATTTAAAACTTTCAACAACGGATCTCTTGGTTCCGGCATCGATGAAGAAC
GCAGCGAAATGCGATACGTAATGTGAATTGCAAATTCAGTGAATCATCGAGTCTTTGAACGCACATTGCGCCC
CCTGGTATTCCGGGGGGCATGCCTGTCCGAGCGTCATTGCTGCCCTCAAGCCCGGCTTGTGTGTTGGGCCCCG
TCCTCCGATTCCGGGGGACGGGCCCGAAAGGCAGCGGCGGCACCGCGTCCGGTCCTCGAGCGTATGGGGCT
TTGTCACCCGCTCTGTAGGCCCGGCCGGCGCTTGCCGATCAACCCAAATTTTTATCCCAGGTGACCTCGGATCA
GGTAGGATACGACAGTAC

>ON844338(F.I)

GGTCACATTCATAAGTTGGGGTTTTAC

GGCGTGGCCGCGACGATTACCAATAACGAGGTGTATGATTACCACACTAG

GAAGCTCGACGTGACCGCCCATCGATTTGGGGAACGCGGGTTACCGCGAG

TCCCAACACCAAGCTGAGCTTGAGGGTTGAAATGACGCTTGAACAGGCTT

GCCCGCCAGAATACTGGCGGGCGCAATGTGCGTTCAAAGATTCGATGATT

CACTGAATTCTGCAATTCACATTACTTATCGCATTTTGCTGCGTTCTTCA

TCGATGCCTAACCCAAGAGATCCGTTGTTGAAAGTTTTGATTTATTTGTT

TGTTTTACTCAGAAGTTCCACTAAAAACAGAGTTTAGGGGTCCTCGGGCG

GGCCGTCCCTTTTTACGGGGCGCGGGCTGATCCGCCGAGGCAACGTATAG

GTATGTTCACAGGGGTTTGGGAGTTGTAAACTCGGTAATGATCCCTCCGC

>MG543801(F.I)
ATATGCTTAAGTTCAGCGGGTATTCCTACCTGATCCGAGGTCACATTCAGAAGTTGGGGTTTTACGGCGTGGCCG
CGACGATTACCAGTAACGAGGTGTATGATTACTACGCTATGGAAGCTCGACGTGACCGCCAATCGATTTGGGGAA
CGCGGGTTACCGCGAGTCCCAACACCAAGCTGAGCTTGAGGGTTGAAATGACGCTCGAACAGGCATGCCCGCCAG
AATACTGGCGGGCGCAATGTGCGTTCAAAGATTCGATGATTCACTGAATTCTGCAATTCACATTACTTATCGCAT
TTTGCTGCGTTCTTCATCGATGCCAGAACCAAGAGATCCGTTGTTGAAAGTTTTGATTTATTTGTTTGTTTTACT
CAGAAGTTCCACTAAAAACAGAGTTTAGGGGTCCTCGGGCGGGCCGTCCCTTTTTACGGGGCGCGGGCTGATCCG
CCGAGGCAACGTATAGGTATGTTCACAGGGGTTTGGGAGTTGTAAACTCGGTAATGATCCCTCCGCAG

>BHU118(Ascomycota.sp)

TGATCCGAGGTCACCTGGATGAAAGTTTGGTGTGATA

TCGCCGGCGCCCGGCAGGCCTACCGAACGGGGGGAAAACCCCCAACCCCT

GAAGAACGGAAGCCGTGCCCCCCCTTCCCTTTCGGGCCCTCCCCCCGGAA

AGAAGAAAGAACCCAACCCACAAACCCTGCTTGAAGGGGCCATGACCCTT

GGAAAGGGGCCCCCCGAATACCCCAAGGGGCAATGGGCGTTCAAAGACT

CCATGAATCACTGAATTTTGCAATTTCATTACGTAACGCAATTTCCTGCG

TTCTTCCACCATTCCCGAACCCAAAAATCCCTTTTTTAAAGTTTTAAATA

ATTTAAATTTTGTCTCAAAACAACATC

>OK095335(A.F)

CTTAGAAAAATAAAGTTGGGTGTCGGCTGGCGCCGGCCGGGCCTACAGAGCAGGTGACAAAGCCCCATACGC
TCGAGGACCGGACGCGGTGCCGCCGCTGCCTTTCGGGCCCGTCCCCCGGGAGAGGGGGACGGGGGCCCAAC
ACACAAGCCGTGCTTGAGGGCAGCAATGACGCTCGGACAGGCATGCCCCCCGGAATACCAGGGGGCGCAAT
GTGCGTTCAAAGACTCGATGATTCACTGAATTCTGCAATTCACATTACTTATCGCATTTCGCTGCGTTCTTCATC
GATGCCGGAACCAAGAGATCCGTTGTTGAAAGTTTTAACTGATTACGATAATCAACTCAGACTGCATACTTTCA
GAACAGCGTTCATGTTGGGGTCTTCGGCGGGCGCGGGCCCGGGGGCGCAAGGCCTCCCCGGCGGCCGTCGA
AACGGCGGGCCCGCCGAAGCAACAAGGTACGATAGACACGGGTGGGAGGTTGGACCCAGAGGGCCCTCACT
CGGTAATGATCCTTCCGCAGGTCCACTTACGGAAGATCAACCGANGTGAGGGCCCTCTGGGTCCACCTCCACC
CGTGTCTTCTACTTGTT

>MW857110(P.B)

GTCAGCCTGGAAGAAGTTTGGTATGATCGGCAGGCGCCGGCCAGTCCTACAAAGCGGGTG
ACAAAGCCCCATACGCTTGAGGACCGGACGCGGTGCCGCCGCTGCCTTTCGGGCCCGTCG
ACCGGAAGGAGGACAGAGGGGAACAGAAAGCCGAGCTTGAGGGCATCAATGACGCTCGGA
CAGGCATGCCCTCCGGAATACCAGAGGGCGCAATGTGCGTTCAAAGACTCGATGATTCACT
GAATTCTGCAATTCACATTACGTATCGCATTTCGCTGCGTTCTTCATCGATGCCGGAACCAA
GAGATCCGTTGTTGAAAGTTTTAAATAATTTATATTTAATCTCAGACTACAATCTTCAGACAGA
GTTCTAAGGTGTCTTCGGCGAGCGCGGACCCGGGGACAGACGTCCCCCGGCAGCCAAAAG
GCAGGCTCGCCGAAGCAACAAGGTAAAATAAACACGGGTGGGAGGTTGGACCCAGAGGGC
CCTCACTCGGTAATGATCCTTCCGCAGGTTCACCCTACGGGAGGATCATTACCGAGTGAGG
GCCCTCTGGGTCCACCTCCACCGTGTTTATTTTACCTTGTTGCTTCGGCACCTGCCTTTTGG
CTGCCGGGGGGACGTCTGTCCCGGGTCGCGCTCCGAAAA

>ON845446(H.V)

GACTTCAAGTCTTGTTGGAGAAATGAAAAGGAATT

TATTGCCTAACGCACGTTAACTATTCCTTGGTCTCTCCCTTCAAGCATAA

TCGTTATACTACCAAGTAAATACAGCGTCTCCAGTAATACCTACTGTTGA

AGGAGAAAAAACAGAAACAGTCTCCAATTTCAAGCTTTTTTTGATTATCG

CCCACGACCGAAAAATAATAAATTATCTTTTGAGAAGGAAATGACGCTCA

AACACGCATGCCCCTGAGAATTTTAAAGGGAGCAATGTGAGTTCAAAAAT

TCAATGATTCACGAGTATCTGCAATTCATATTACTTATCGCAATTCGCTA

CGTTCTTCGGCGATGCGAGAACCAAGAGATCCGTTGTTGAAAGTTTTAAA

TTATTTTAAAATTTCCGTTAGGAATTTTGGTTTAGTTTAAAAAATTATAA

TAAAATAAAATTGTTTGTGTTTGTTTTTTGCCTTGAACCTTTCGATTCAA

AGC

>MK613264(H.V)

ACGCCGAACCAGCCATGATTTGAGGTCACACTTGATGAATATTAAAAGCAACCCTTTGCCTAAGGTACGTTAC
CATTTCCCTTGTAAAGTAAAACGAATAAATCCATAAATACATCACAGCGAGAACAGCGTCTCCAAAGAAGCTA
AGTGTTGAATTAAAAAAGACTGAAACAGTCTCCAATTTCAAGCTAACCCTGAGTATCGCCCACAACCAAAAAA
TAATAAATTATCTTTTGAGAAGGAAATGACGCTCAAACAGGCATGCCCCTGAGAATGCTCAAGGGCGCAATGT
GCGTTCAAAAATTCAATGATTCACGAGTATCTGCAATTCACATTACTTATCGCAATTCGCTACGTTCTTCATCGA
TGCGAGAACCAAGAGATCCGTTGTTGAAAGTTTTAAATTATTTTAAAATTTCCGTTAGGAATTTTGGTTTAGTT
TAAAAAATTATAATAAAATAAAATTGTTTGTGTTTGTTTTTTGCCTTGAACCTTTCGATTCAAAGCAGAAAGAAT
TAAATTAAAGTAAAAAACTCCAATGTGTGTAGCGTTGACTGAGATTCAAGCAAGACTACTTTCACTGCGACAC
TCTTAAGAAGCAGCGCAATTAAGCGCACATCTTCAATAAAGATACACATTATTGTAAAAGATCTAAACAAGAA
CTCGAGCAACAATGATTATTCAATCTAATGATCCTTCCGCAGGTACCCCTA

Alighnemten

>ON845652(B.P)

TGATCCGAGGTCAAAGTGAGAAATGGACTTGATGGA

TTGCTGACCTCTTTAGCTGGTTGTAAGCGCAAAAAATGTGCTGCGCTCCG

AAACCAGTAGGCCGGCTGCCAATCGTTTTAAGGCGAGTCTCGCGGCTAGG

CAAGACAAAAAAAACGCCCAACACCAGGCAAAGCTTGAGGGTACAAATGA

CGCTGGAACAGGCATGCCCTTTGGAATACCAAAGGGCGCAATGTGCGTTC

AAAGATTCGATGATTCACTGAATTCTGCAATTCACACTACGTATCGCATT

TCGCTGCGTTCTTCATCGATGCCGGAACCGAGAGATCCATTGTTGAAAGT

TGTAAATGATTTAACATTTGTTATACTGACGCTTATTGCAACTACTAAAA

AAAGGTTTATGGTGTGGTCCTGGTGGCGGGCGAACCCGCCCAGGAAACAA

CAAGTACTCATAACACTTGGGTGGAAAAATAGTTCACCCTCCGCCACTGT

>KY950237(B.P)

AGTATGCTTAAGTTCAGCGGGTATCCCTACCTGATCCGAGGTCAAAAGTGAGAAATGGACTTGATGGATTGCT
GACCTCTTTAGCTGGTTGTAAGCGCAAAAAATGTGCTGCGCTCCGAAACCAGTAGGCCGGCTGCCAATCGTTT
TAAGGCGAGTCTCGCGGCAAGGCAAGACAAAAAAAACGCCCAACACCAAGCAAAGCTTGAGGGTACAAATG
ACGCTCGAACAGGCATGCCCTTTGGAATACCAAAGGGCGCAATGTGCGTTCAAAGATTCGATGATTCACTGAA
TTCTGCAATTCACACTACGTATCGCATTTCGCTGCGTTCTTCATCGATGCCAGAACCAAGAGATCCGTTGTTGA
AAGTTGTAAATGATTTAACATTTGTTATACTGACGCTGATTGCAACTACATAAAAAAAGGTTTATGGTGTGGTC
CTGGTGGCGGGCGAACCCGCCCAGGAAACAACAAGTACGCAAAAGACATGGGTGGAAAAATAGTTCAGCCT
CCGCCACTGTGCCCGGAGGCGCCAGCGCATACAGCCTTCATATTTTTTGTGAA

>ON845732(A.T)

CAAGTTGCAAATAAATGCGTCGGCGGGCGCCGGCCGGGCCTACGGAGCGGAAGACGAAGCC

CCATACGCTCGAGGACCGGACGCGGTGCCGCCGCTGCCTTTCGGGCCCGT

CCCCCGGGAGCCGGGGGACGAGGGCCCAACACACAAGCCGGGCTTGAGGG

CAGCAATGACGCTCGGACAGGCATGCCCCCCGGAATACCAGGGGGCGCAA

TGTGCGTTCAAAGACTCGATGATTCACTGAATTCTGCAATTCACATTAGT

TATCGCATTTCGCTGCGTTCTTCATCGATGCCGGAACCAAGAGATCCATT

GTTGAAAGTTTTAACTGATTGCAAAGAATCACACTCAGACTGCAAGCTTT

CAGAACAGGGTTCATGTTGGGGTCTCCGGCGGGCACGGGCCCGGGGGCGA

GTCGCCCCCCGGCGGCCAGCAACGCTGGCGGGCCCGCCGAAGCAACAAGG

TACAATAGTCACGGGTGGGGGAGGGTTGGGCCATAAAGACCCGCACTCGG

TAATGATCCTTCCGCAACACCCCCCTAAAAGAGAGGTCAAT

>KY684268(A.T)

ATGATATGCTTAAGTTCAGCGGGTATCCCTACCTGATCCGAGGTCAACCTGGAAAAAAACAAGTTGCAAATAA
ATGCGTCGGCGGGCGCCGGCCGGGCCTACGGAGCGGAAGACGAAGCCCCATACGCTCGAGGACCGGACGC
GGTGCCGCCGCTGCCTTTCGGGCCCGTCCCCCGGGAGCCGGGGGACGAGGGCCCAACACACAAGCCGGGCT
TGAGGGCAGCAATGACGCTCGGACAGGCATGCCCCCCGGAATACCAGGGGGCGCAATGTGCGTTCAAAGAC
TCGATGATTCACTGAATTCTGCAATTCACATTAGTTATCGCATTTCGCTGCGTTCTTCATCGATGCCGGAACCAA
GAGATCCATTGTTGAAAGTTTTAACTGATTGCAAAGAATCACACTCAGACTGCAAGCTTTCAGAACAGGGTTC
ATGTTGGGGTCTCCGGCGGGCACGGGCCCGGGGGCGAGTCGCCCCCCGGCGGCCAGCAACGCTGGCGGGCC
CGCCGAAGCAACAAGGTACAATAGTCACGGGTGGGGAGGTTGGGCCATAAAGACCCGCACTCGGTAATGAT
CCTTCCGCA

>ON845734(F.O)

GATTCGAGGTCAACATTCAGAAGTTGGGTGTTT

TACGGCATGGCCGCGCCGCTTCCAGTTGCGAGGTGTTAGCTACTACGCAT

TGGAAGCTGCGGCGGGACCGCCCTGTATTTGAGGGACGGCGTGTGCCCAC

AGGGGGCTTCTGCCGATCCCCAACGCCGGCCCGGGGGCCTGAGGGTTGTA

ATGACGCTCGAACAGGCATGCCCGCCAGAATACTGGCGGGCGCAATGTGC

GTTCAAAGATTCGATGATTCACTGAATTCTGCAATTCACATTACTTATCG

CATTTCGCTGCGTTCTTCATCGATGCCTGAGCCTAGAGATCCGTTGTTGA

AAGTTTTAATTTATTTGCTTGTTTACTCAAAAAAACATTATAAAAACAGA

GTTAGGGGTCCTCTGGCGGGGGCGGCCCGTTGTTACAGGGCCGTCTGTTC

CCGCCGAAGCAACGTTTTAGGTATGTTCACAGGGTTGATGAGTTGTATAA

CTCGGTAATGATCCCTCC

>MT967273(F.O)

GCATTCTACCTGATTCGAGGTCAACATTCAGAAGTTGGGTGTTTTACGGCATGGCCGCGCCGCTCTCCAGTTG
CGAGGTGTTAGCTACTACGCAATGGAAGCTGCGGCGGGACCGCCACTGTATTTGAGGGACGGCGTGTGCCCA
CAGGGGGCTTCTGCCGATCCCCAACGCCAGGCCCGGGGGCCTGAGGGTTGTAATGACGCTCGAACAGGCATG
CCCGCCAGAATACTGGCGGGCGCAATGTGCGTTCAAAGATTCGATGATTCACTGAATTCTGCAATTCACATTA
CTTATCGCATTTCGCTGCGTTCTTCATCGATGCCAGAGCCAAGAGATCCGTTGTTGAAAGTTTTAATTTATTTGC
TTGTTTACTCAGAAAAACATTATAAAAACAGAGTTAGGGGTCCTCTGGCGGGGGCGGCCCGTTGTTACAGGG
CCGTCTGTTCCCGCCGAAGCAACGTTTTAGGTATGTTCACAGGGTTGATGAGTTGTATAACTCGGTAATGATCC
CTCCGCAGGCCCCCCCTACAGAAAAG

>ON866891(A.N)

AGGTCACCTGGAAGAATGGTTGGAAA

ACGTCGGCGGCGCCGGCCAATCCTACGAGCATGTGACAAAGCCCCATACG

CTCGAGGATCGGACGCGGTGCCGCCGCTGCCTTTCGGGCCCGTCCCCCCG

GAGAGGGGGACGGCGACCCAACACACAAGCCGGGCTTGAGGGCAGCAATG

ACGCTTGGACAGGCATGCCCCCCGGAATACCAGGGGGCGCAATGTGCGTT

CAAAGACTCGATGATTCACTGAATTCTGCAATTCACATTAGTTATCGCAT

TTCGCTGCGTTCTTCATCGATGCCGGAACCAAGAGATCCATTGTTGAAAG

TTTTAACTGATTGCATTCAATCAACTCAGACTGCACGCTTTCAGACAGTG

TTCGTGTTGGGGTCTCCGGCGGGCACGGGCCCGGGGGGCAAAGGCGCCCC

CCCGGCGGCCGACAAGCGGCGGGCCCGCCGAAGCAACAGGGTACAATAGA

>MH091026(A.N)

TTCCTCCGCTTATTGATATGCTTAAGTTCAGCGGGTATCCCTACCTGATCCGAGGTCACCTGGAAAGAATGGTT
GGAAAACGTCGGCAGGCGCCGGCCAATCCTACAGAGCATGTGACAAAGCCCCATACGCTCGAGGATCGGAC
GCGGTGCCGCCGCTGCCTTTCGGGCCCGTCCCCCCGGAGAGGGGGACGGCGACCCAACACACAAGCCGGGC
TTGAGGGCAGCAATGACGCTCGGACAGGCATGCCCCCCGGAATACCAGGGGGCGCAATGTGCGTTCAAAGA
CTCGATGATTCACTGAATTCTGCAATTCACATTAGTTATCGCATTTCGCTGCGTTCTTCATCGATGCCGGAACCA
AGAGATCCATTGTTGAAAGTTTTAACTGATTGCATTCAATCAACTCAGACTGCACGCTTTCAGACAGTGTTCGT
GTTGGGGTCTCCGGCGGGCACGGGCCCGGGGGGCAGAGGCGCCCCCCGGCGGCCGACAAGCGGCGGGCC
CGCCGAAGCAACAGGGTACAATAGACACGGATGGGAGGTTGGGCCCAAAGGACCCGCACTCGGTAATGATC
CTTCCGCAGGTTCACCTACGGAAGGATCATTACNGAGTGCGGGTCCTTTGGGCCCAACCTCCCATCCGTGTCT
ATTGTACCCTGTTGCTTCGGCGGGCCCGCCGCTTGTCGGCCGCCGGGGGG

>ON834317(A.N)

AGGTCAACCTGGAAGAATGGTTGGAAAA

CGTCGGCAGGCGCCGGCCAATCCTACAGAGCATGTGACAAAGCCCCATAC

GCTCGAGGATCGGACGCGGTGCCGCCGCTGCCTTTCGGGCCCGTCCCCCC

GGAGAGGGGGACGGCGACCCAACACACAAGCCGGGCTTGAGGGCAGCAAT

GACGCTCGGACAGGCATGCCCCCCGGAATACCAGGGGGCGCAATGTGCGT

TCAAAGACTCGATGATTCACTGAATTCTGCAATTCACATTAGTTATCGCA

TTTCGCTGCGTTCTTCATCGATGCCGGAACCAAGAGATCCATTGTTGAAA

GTTTTAACTGATTGCATTCAATCAACTCAGACTGCACGCTTTCAGACAGT

GTTCGTGTTGGGGTCTCCGGCGGGCACGGGCCCGGGGGGCAGAGGCGCCC

CCCCGGCGGCCGACAAGCGGCGGGCCCGCCGAAGCAACAGGGTACAATAG

ACACGGATGGGGAGGGTTGGGCCCAAAGGACCCGCACTCGGTAATGATCC

TTCCGCA

>ON241768(A.N)

CCTACCTGATCCGAGGTCAACCTGGAAAGAATGGTTGGAAAACGTCGGCAGGCGCCGGCCAATCCTACAGAG
CATGTGACAAAGCCCCATACGCTCGAGGATCGGACGCGGTGCCGCCGCTGCCTTTCGGGCCCGTCCCCCCGG
AGAGGGGGACGGCGACCCAACACACAAGCCGGGCTTGAGGGCAGCAATGACGCTCGGACAGGCATGCCCCC

CGGAATACCAGGGGGCGCAATGTGCGTTCAAAGACTCGATGATTCACTGAATTCTGCAATTCACATTAGTTAT
CGCATTTCGCTGCGTTCTTCATCGATGCCGGAACCAAGAGATCCATTGTTGAAAGTTTTAACTGATTGCATTCA
ATCAACTCAGACTGCACGCTTTCAGACAGTGTTCGTGTTGGGGTCTCCGGCGGGCACGGGCCCGGGGGGCAG
AGGCGCCCCCCCGGCGGCCGACAAGCGGCGGGCCCGCCGAAGCAACAGGGTACAATAGACACGGATGGGA
GGTTGGCCCCAAAGGACCCGCACTCGGTAATGATCCTTCCGCAGGTTCACCTACGGAAGGATCATTACCGAGT
GCGGGTCCTTTGGGCCCAACCTCCCATCCGTGTCTATTGTACCCTGTTGCTTCGGCGGGCCCGCCGCTTGTCGG
CCGCCGGGGGGGCGCCTCTGCCCCCCGGGCCCGTGCCCGCCGGAGACCCCAACACGAACACTGTCTGAAAGC
GTGCAGTCTGAGTTGATTGAATGCAATCAGTTAAAACTTTCAACAATGGATCTCTTGGTTCCGGCATCGATGAA
GAACGCAGCGAAATGCGATAACTAATGTGAATTGCAGAATTCAGTGAATCATCGAGTCTTTGAACGCACATTG
CGCCCCCTGGTATTCCGGGGGGCATGCCTGTCCGAGCGTCATTGCTGCCCTCAAGCCCGGCTTGTGTGTTGGG
TCGCCGTCCCCCTCTCCGGGGGGACGGGCCCGAAAGGCAGCGGGGGCACCGGCGTCCGATCCTCGAGCGTTT
GGGGCTTTGTCACATGCTCCGTAGGATTGGCCGGCGCCTGCCGACGTTTTCCAACCATTCTTTCCAGGTTGACC

>ON847316(A.P)

ATCCGAGGTCACCTGAGAAAAATAAGGTTGG

AGACGCCGGCTGGCGCCCGGCCGGCCCTAATCGAGCGGGTGACAAAGCCC

CATACGCTCGAGGACCGGACACGGTGCCGCCGCTGCCTTTCGGGCCCGTC

CCCCGGGGGGGACGACGACCCAACACACAAGCCGGGCTTGAGGGCAGCAA

TGACGCTCGGACAGGCATGCCCCCCGGAATGCCAGGGGGCGCAATGTGCG

TTCAAAGACTCGATGATTCACTGAATTCTGCAATTCACATTACTTATCGC

AGTTCGCTGCGTTCTTCATCGATGCCGGAACCAAGAGATCCATTGTTGAA

AGTTTTGACTGATTTGTATTCAGGCTCAGACTGCATCACTCTCAGGCATG

AAGTTCAGTGG

>MT712158(A.P)

AGTTTGATCCATTCCTGATCCGAGGTCAACCTGAGAAAAATAAGGTTGGAGACGCCGGCTGGCGCCCGGCCG
GCCCTAATCGAGCGGGTGACAAAGCCCCATACGCTCGAGGACCGGACACGGTGCCGCCGCTGCCTTTCGGGC
CCGTCCCCCGGGGGGGACGACGACCCAACACACAAGCCGGGCTTGAGGGCAGCAATGACGCTCGGACAGGC
ATGCCCCCCGGAATGCCAGGGGGCGCAATGTGCGTTCAAAGACTCGATGATTCACTGAATTCTGCAATTCACA
TTACTTATCGCAGTTCGCTGCGTTCTTCATCGATGCCGGAACCAAGAGATCCATTGTTGAAAGTTTTGACTGAT
TTGTATTCAGGCTCAGACTGCATCACTCTCAGGCATGAAGTTCAGTGGTCCCCGGCGGCTCGCCCCTAGGGGG
CTCCCCGCCGAAGCAACAGTGTTAGGTAGTCACGGGTGGGAGGTTGGGCGCCCGGAGGCAGCCCGCACTCG
GCAATGATCCTTCCGCAGGTTCCCCTACGGAAGG

>ON866737(A.N)

ATCCGAGGTCAACCTGGAAAGAATGGTTG

GAAAACGTCGGCCGGCGCCGGCCAATCCTACAGAGCATGTGACAAAGCCC

CATACGCTCGAGGATCGGACGCGGTGCCGCCGCTGCCTTTCGGGCCCGTC

CCCCCGGAGAGGGGGACGGCGACCCAACACACAAGCCGGGCTTGAGGGCA

GCAATGACGCTCGGACAGGCATGCCCCCCGGAATACCAGGGGGCGCAATG

TGCGTTCAAAGACTCGATGATTCACTGAATTCTGCAATTCACATTAGTTA

TCGCATTTCGCTGCGTTCTTCATCGATGCCGGAACCAAGAGATCCATTGT

TGAAAGTTTTAACTGATTGCATTCAATCAACTCAGACTGCACGCTTTCAG

ACAGTGTTCGTGTTGGGGTCTCCGGCGGGCACGGGCCCGGGGGGCAGAGG

CGCCCCCCCGGCGGCCGACAAGCGGCGGGCCCGCCGAAGCAACAGGGTAC

AATAGACACGGATGGGAGGTTGGGCCCAAAGGACCCGCACTCGGTAATGA

TCCTTCCGCAGGTCAACCCTACAGAAGATCATTACCGAGTGCGGGTCCTT

TGGGCCCACCTCCCATCCTGTCTATGT

>ON847317(A.T)

GATCCGAGGTCAACCTGGAAAAAATGGTTGGAAA

ACGTCGGCGGCGCCGGCCAATCCTACAGAGCATGTGACAAAGCCCCATAC

GCTCGAGGATCGGACGCGGTGCCGCCGCTGCCTTTCGGGCCCGTCCCCCC

GGAGAGGGGGACGGCGACCCAACACACAAGCCGGGCTTGAGGGCAGCAAT

GACGCTCGGACAGGCATGCCCCCCGGAATACCAGGGGGCGCAATGTGCGT

TCAAAGACTCGATGATTCACTGAATTCTGCAATTCACATTAGTTATCGCA

TTTCGCTGCGTTCTTCATCGATGCCGGAACCAAGAGATCCATTGTTGAAA

GTTTTAACTGATTGCATTCAATCAACTCAGACTGCACGCTTTCAGACAGT

GTTCGTGTTGGGGTCTCCGGCGGGCACGGGCCCGGGGGGCAAAGGCGCCC

CCCCGGCGGCCGACAAGCGGCGGGCCCGCCGAAGCAACAGGGTATAATAG

ACACGGATGGGAGGTTGGGCCCAAAGGACCCGCACTCGGTAATGATCCTT

CCGCAGCACCCCCTCTCAGAGAAAGAA

>MT446141(A.T)

AGGATGGGCTCTACCTGATCCGAGGTCACCTGGAAAAAATGGTTGGAAAACGTCGGCAGGCGCCGGCCAATC
CTACAGAGCATGTGACAAAGCCCCATACGCTCGAGGATCGGACGCGGTGCCGCCGCTGCCTTTCGGGCCCGT
CCCCCCGGAGAGGGGGACGGCGACCCAACACACAAGCCGGGCTTGAGGGCAGCAATGACGCTCGGACAGGC
ATGCCCCCCGGAATACCAGGGGGCGCAATGTGCGTTCAAAGACTCGATGATTCACTGAATTCTGCAATTCACA
TTAGTTATCGCATTTCGCTGCGTTCTTCATCGATGCCGGAACCAAGAGATCCATTGTTGAAAGTTTTAACTGAT
TGCATTCAATCAACTCAGACTGCACGCTTTCAGACAGTGTTCGTGTTGGGGTCTCCGGCGGGCACGGGCCCGG
GGGGCAAAGGCGCCCCCCCGGCGGCCGACAAGCGGCGGGCCCGCCGAAGCAACAGGGTATAATAGACACG
GATGGGAGGTTGGGCCCAAAGGACCCGCACTCGGTAATGATCCTTCCGCAGGTTCACCTACGGAAACCTTGTT
ACGACTTTTACTTCCTCTAAATGGACCAAGA

>ON866738(A.N)

GATCCGAGGTCACCTGGAAAGAATGGTTGGAA

AACGTCGGCGGCGCCGGCCAATCCTACAGAGCATGTGACAAAGCCCCATA

CGCTCGAGGATCGGACGCGGTGCCGCCGCTGCCTTTCGGGCCCGTCCCCC

CGGAGAGGGGGACGGCGACCCAACACACAAGCCGGGCTTGAGGGCAGCAA

TGACGCTCGGACAGGCATGCCCCCCGGAATACCAGGGGGCGCAATGTGCG

TTCAAAGACTCGATGATTCACTGAATTCTGCAATTCACATTAGTTATCGC

ATTTCGCTGCGTTCTTCATCGATGCCGGAACCAAGAGATCCATTGTTGAA

AGTTTTAACTGATTGCATTCAATCAACTCAGACTGCACGCTTTCAGACAG

TGTTCGTGTTGGGGTCTCCGGCGGGCACGGGCCCGGGGGGCAGAGGCGCC

CCCCCGGCGGCCGACAAGCGGCGGGCCCGCCGAAGCAACAGGGTACAATA

GACACGGATGGGGGAGGTTGGGCCCAAAGGACCCGCACTCGGTAATGATC

CTTCCGCATCCCCCCCCCTACAGAGAGGATCATTACCGAGTGCGGGTCCT

TGGGCCCACCTCCCACCTGTCTATG

>MF078659(A.N)

GCGGGCACGGCCCGGGGGCAAGGCGCCCCCCGGCGGCCGACAGCGGCGGGCCCGCCGAAGCAACAGGGTA
TATAGACAGGATGGGAGGTTGGGCCCAAAGGACCCGCACTCGGTAATGATTCCTCCGGCCTATTGATATGCTT
AAGTTCAGCGGGTATCCCTACCTGATCCGAGGTCAACCTGGAAAAAATGGTTGGAAAACGTCGGCAGGCGCC
GGCCAATCCTACAGAGCATGTGACAAAGCCCCATACGCTCGAGGATCGGACGCGGTGCCGCCGCTGCCTTTC
GGGCCCGTCCCCCCGGAGAGGGGGACGGCGACCCAACACACAAGCCGGGCTTGAGGGCAGCAATGACGCTC
GGACAGGCATGCCCCCCGGAATACCAGGGGGCGCAATGTGCGTTCAAAGACTCGATGATTCACTGAATTCTG
CAATTCACATTAGTTATCGCATTTCGCTGCGTTCTTCATCGATGCCGGAACCAAGAGATCCATTGTTGAAAGTT
TTAACTGATTGCATTCAATCAACTCAGACTGCACGCTTTCAGACAGTGTTCGTGTTGGGGTCTCCGGCGGGCA
CGGGCCCGGGGGGCAAAGGCGCCCCCCGGCGGCCGACAAGCGGCGGGCCCGCCGAAGCAACAGGGTATA
ATAGACACGGATGGGAGGTTGGGCCCAAAGGACCCGCACTCGGTAATGATCCTTCCGCAGGTCACCCTACGG
AAGCATTACCGAGTGCGGGTCCTTGGGCCCAACCTCCCATCCTGTCTATATACCCGTTGCTCGGCGGGCGCCC
TGTCGGCGCCGGGGGGCGCTTGCCCCGGGCCGTGCGCGA

>ON845807(P.V)

GATCCGAGGTCAACCTGGATAAAAATTTGGGTTGATC

GGCAAGCGCCGGCCGGGCCTACAGAGCGGGTGACAAAGCCCCATACGCTC

GAGGACCGGACGCGGTGCCGCCGCTGCCTTTCGGGCCCGTCCCCCGGAAT

CGGAGGACGGGGCCCAACACACAAGCCGTGCTTGAGGGCAGCAATGACGC

TCGGACAGGCATGCCCCCCGGAATACCAGGGGGCGCAATGTGCGTTCAAA

GACTCGATGATTCACTGAATTTGCAATTCACATTACGTATCGCATTTCGC

TGCGTTCTTCATCGATGCCGGAACCAAGAGATCCGTTGTTGAAAGTTTTA

AATAATTTATATTTTCACTCAGACTTCAATCTTCAGACAGAGTTCGAGGG

TGTCTTCGGCGGGCGCGGGCCCGGGGGCGTGAGCCCCCCGGCGGCCAGTT

AAGGCGGGCCCGCCGAAGCAACAAGGTAAAATAAACACGGGTGGGAGGTT

GGACCCAAAGGGCCCTCACTCGGTAATGATCCTTCCG

>MK583349(P.V)

GGGGCAATCCCTGTTGGTTTCTTTTCCTCCGCTTATTGATATGCTTAAGTTCAGCGGGTATCCCTACCTGATCCG
AGGTCAACCTGGATAAAAATTTGGGTTGATCGGCAAGCGCCGGCCGGGCCTACAGAGCGGGTGACAAAGCC
CCATACGCTCGAGGACCGGACGCGGTGCCGCCGCTGCCTTTCGGGCCCGTCCCCCGGAATCGGAGGACGGGG
CCCAACACACAAGCCGGGCTTGAGGGCAGCAATGACGCTCGGACAGGCATGCCCCCCGGAATACCAGGGGG
CGCAATGTGCGTTCAAAGACTCGATGATTCACTGAATTTGCAATTCACATTACGTATCGCATTTCGCTGCGTTC
TTCATCGATGCCGGAACCAAGAGATCCGTTGTTGAAAGTTTTAAATAATTTATATTTTCACTCAGACTTCAATCT
TCAGACAGAGTTCGAGGGTGTCTTCGGCGGGCGCGGGCCCGGGGGCGTGAGCCCCCCGGCGGCCAGTAAAG
GCGGGCCCGCCGAAGCAACAAGGTAAAATAAACACGGGTGGGAGGTTGGACCCAAAGGGCCCTCACTCGGT
AATGATCCTTCCGCAGGTTCACCTACGGA

>ON833481(A.T)

GTTGCAAATAAATGCGTCGGCGGGCGCCGGCCGGGCCTACGGAGCGGAAGACGAA

GCCCCATACGCTCGAGGACCGGACGCGGTGCCGCCGCTGCCTTTCGGGCC

CGTCCCCCGGGAGCCGGGGGACGAGGGCCCAACACACAAGCCGGGCTTGA

GGGCAGCAATGACGCTCGGACAGGCATGCCCCCCGGAATACCAGGGGGCG

CAATGTGCGTTCAAAGACTCGATGATTCACTGAATTCTGCAATTCACATT

AGTTATCGCATTTCGCTGCGTTCTTCATCGATGCCGGAACCAAGAGATCC

ATTGTTGAAAGTTTTAACTGATTGCAAAGAATCACACTCAGACTGCAAGC

TTTCAGAACAGGGTTCATGTTGGGGTCTCCGGCGGGCACGGGCCCGGGGG

CGAGTCGCCCCCCGGCGGCCAGCAACGCTGGCGGGCCCGCCGAAGCAACA

AGGTACAATAGTCACGGGTGGGAGGTTGGGCCATAAAGACCCGCACTCGG

TAATGATCCTTCCGCAGGT

>KY684270(A.T)

TATTGATATGCTTAAGTTCAGCGGGTATCCCTACCTGATCCGAGGTCAACCTGGAAAAAAACAAGTTGCAAAT
AAATGCGTCGGCGGGCGCCGGCCGGGCCTACGGAGCGGAAGACGAAGCCCCATACGCTCGAGGACCGGAC
GCGGTGCCGCCGCTGCCTTTCGGGCCCGTCCCCCGGGAGCCGGGGGACGAGGGCCCAACACACAAGCCGGG
CTTGAGGGCAGCAATGACGCTCGGACAGGCATGCCCCCCGGAATACCAGGGGGCGCAATGTGCGTTCAAAG
ACTCGATGATTCACTGAATTCTGCAATTCACATTAGTTATCGCATTTCGCTGCGTTCTTCATCGATGCCGGAACC
AAGAGATCCATTGTTGAAAGTTTTAACTGATTGCAAAGAATCACACTCAGACTGCAAGCTTTCAGAACAGGGT
TCATGTTGGGGTCTCCGGCGGGCACGGGCCCGGGGGCGAGTCGCCCCCCGGCGGCCAGCAACGCTGGCGGG
CCCGCCGAAGCAACAAGGTACAATAGTCACGGGTGGGAGGTTGGGCCATAAAGACCCGCACTCGGTAATGAT
CCTTCCGCAGGTCACCCTACGG

>ON834321(ACT.ELE)

ATCCGAGGTCACCTGGAAAAATGGTTGGAAAACG

TCGGCAGGCGCCGGCCAATCCTACAGAGCATGTGACAAAGCCCCATACGC

TCGAGGATCGGACGCGGTGCCGCCGCTGCCTTTCGGGCCCGTCCCCCCGG

AGAGGGGGACGGCGACCCAACACACAAGCCGGGCTTGAGGGCAGCAATGA

CGCTCGGACAGGCATGCCCCCCGGAATACCAGGGGGCGCAATGTGCGTTC

AAAGACTCGATGATTCACTGAATTCTGCAATTCACATTAGTTATCGCATT

TCGCTGCGTTCTTCATCGATGCCGGAACCAAGAGATCCATTGTTGAAAGT

TTTAACTGATTGCATTCAATCAACTCAGACTGCACGCTTTCAGACAGTGT

TCGTGTTGGGGTCTCCGGCGGGCACGGGCCCGGGGGGCAAAGGCGCCCCC

CCGGCGGCCGACAAGCGGCGGGCCCGCCGAAGCAACAGGGTATAATAGAC

ACGGATGGGGAGGTTGGGCCCAAAGGACCCGCACTCGGTAATGATCCTTC

CGCAGC

>MT503300(ACT.ELE)

TTCCTCCCCGCTTTTTTGATATGCTTAAGTTCAGCGGGTATCCCTACCTGATCCGAGGTCAACCTGGAAAAAAT
GGTTGGAAAACGTCGGCAGGCGCCGGCCAATCCTACAGAGCATGTGACAAAGCCCCATACGCTCGAGGATCG
GACGCGGTGCCGCCGCTGCCTTTCGGGCCCGTCCCCCCGGAGAGGGGGACGGCGACCCAACACACAAGCCG
GGCTTGAGGGCAGCAATGACGCTCGGACAGGCATGCCCCCCGGAATACCAGGGGGCGCAATGTGCGTTCAA
AGACTCGATGATTCACTGAATTCTGCAATTCACATTAGTTATCGCATTTCGCTGCGTTCTTCATCGATGCCGGA
ACCAAGAGATCCATTGTTGAAAGTTTTAACTGATTGCATTCAATCAACTCAGACTGCACGCTTTCAGACAGTGT
TCGTGTTGGGGTCTCCGGCGGGCACGGGCCCGGGGGGCAAAGGCGCCCCCCCGGCGGCCGACAAGCGGCG
GGCCCGCCGAAGCAACAGGGTATAATAGACACGGATGGGAGGTTGGGCCCAAAGGACCCGCACTCGGTAAT
GATCCTTCCGCAGGCACCCCCCTAAACGAAAG

>ON868703(A.T)

ACAAGTTGCAAATAAATGCGTCGGCGGGCGCCGGCCGGGCCTACGGAGCGGAAGACGAAGC

CCCATACGCTCGAGGACCGGACGCGGTGCCGCCGCTGCCTTTCGGGCCCG

TCCCCCGGGAGCCGGGGGACGAGGGCCCAACACACAAGCCGGGCTTGAGG

GCAGCAATGACGCTCGGACAGGCATGCCCCCCGGAATACCAGGGGGCGCA

ATGTGCGTTCAAAGACTCGATGATTCACTGAATTCTGCAATTCACATTAG

TTATCGCATTTCGCTGCGTTCTTCATCGATGCCGGAACCAAGAGATCCAT

TGTTGAAAGTTTTAACTGATTGCAAAGAATCACACTCAGACTGCAAGCTT

TCAGAACAGGGTTCATGTTGGGGTCTCCGGCGGGCACGGGCCCGGGGGCG

AGTCGCCCCCCGGCGGCCAGCAACGCTGGCGGGCCCGCCGAAGCAACAAG

GTACAATAGTCACGGGTGGGGAGGTTGGGCCATAAAGACCCGCACTCGGT

AATGATCCTTCCGCAG

>ON844330(ALT.ALT)

CTGATCGAGGTCAAAGTTGAAAAGGCTTAATGGATGCTA

GACCTTTGCTGATAGAGAGTGCGACTTGTGCTGCGCTCCGAAACCAGTAG

GCCGGCTGCCAATTACTTTAAGGCGAGTCTCCAGCAAAGCTAGAGACAAG

ACGCCCAACACCAAGCAAAGCTTGAGGGTACAAATGACGCTCGAACAGGC

ATGCCCTTTGGAATACCAAAGGGCGCAATGTGCGTTCAAAGATTCGATGA

TTCACTGAATTCTGCAATTCACACTACTTATCGCATTTCGCTGCGTTCTT

CATCGATGCCAGAACCAAGAGATCCGTTGTTGAAAGTTGTAATTATTAAT

TTGTTACTGACGCTGATTGCAATTACAAAAGGTTTATGTTTGTCCTAGTG

GTGGGCGAACCCACCAAGGAAACAAGAAGTACGCAAAAGACAAGGGTGAA

TAATTCAGCAAGGCTGTAACCCCGAGAGGTTCCAGCCCGCCTTCATATTT

GTGTAATGATCCCTCCGCAT

>MZ664272(ALT.ALT)

GGGGGGATCTACTGATCCGAGGTCAAAGTTGAAAAAAAGGCTTAATGGATGCTAGACCTTTGCTGATAGAGA
GTGCGACTTGTGCTGCGCTCCGAAACCAGTAGGCCGGCTGCCAATTACTTTAAGGCGAGTCTCCAGCAAAGCT
AGAGACAAGACGCCCAACACCAAGCAAAGCTTGAGGGTACAAATGACGCTCGAACAGGCATGCCCTTTGGAA
TACCAAAGGGCGCAATGTGCGTTCAAAGATTCGATGATTCACTGAATTCTGCAATTCACACTACTTATCGCATT
TCGCTGCGTTCTTCATCGATGCCAGAACCAAGAGATCCGTTGTTGAAAGTTGTAATTATTAATTTGTTACTGAC
GCTGATTGCAATTACAAAAGGTTTATGTTTGTCCTAGTGGTGGGCGAACCCACCAAGGAAACAAGAAGTACG
CAAAAGACAAGGGTGAATAATTCAGCAAGGCTGTAACCCCGAGAGGTTCCAGCCCGCCTTCATATTTGTGTAA
TGATCCCTCCGCAGGTCCCCCTACGGAAG

>ON868765(G.C)

GATCTGAGGTTGAATAGTGTTGTTTTTCAAACG

AATTTGATTCGATTTTTTAGAAAAGCAATGCAATTCCAAGAGAGAAACAA

CGCTCAAACAAGTATACTTTGGGGGATACCCCAAAGTGCAATGTGCGTTC

AAAAACTGATGATTCACTTCTGCAATTCACAAGAAATATCGCGTTTCGCT

GCGTTCTTCATCGATACGAGAACCAAGAGATCCATTGTTAAAAGTTTTGA

TTATTTTTGTTTTGATTGTATGATTATTGTTTGCTGTGGTAATTTCACAA

ATATTTTCAATTCTTAATGATCCTTCCGCAGGTCACCCTACGGAAAAGGA

TCATTAAGAATTGAAAATATTTGTGAAATTACCACAGCAAACAATAATCA

TACTATCAAAACAAAAATAATCAAAACTTTTAACAATGGATCTCTTGGTT

CTCGTATCGA

>MT946913(G.C)

AGCTGCCCTTCCTCCGCTTATTGATATGCTTAAGTTCAGCGGGTAATCCTACTTGATCTGAGGTTGAATAGTGT
TGTTTTTCAAACGAATTTGATTCGAAATTTTAGAAAAGCAATGCAATTCCAAGAGAGAAACAACGCTCAAACA
AGTATACTTTGGGGGATACCCCAAAGTGCAATGTGCGTTCAAAAACTGATGATTCACTTCTGCAATTCACAAG
AAATATCGCGTTTCGCTGCGTTCTTCATCGATACGAGAACCAAGAGATCCATTGTTAAAAGTTTTGATTATTTTT

GTTTTGATTGTATGATTATTGTTTGCTGTGGTAATTTCACAAATATTTATAATTCTTAATGATCCTTCCGCAGGTT
CACCTACGGAAAGGGCAGCT

>ON868706(G.C)

TGATCTGAGGTTGATAGTGTTGTTTTTCAAACGAA

TTTGATTCGATTTTTTAGAAAAGCAATGCAATTCCAAGAGAGAAACAACG

CTCAAACAAGTATACTTTGGGGGATACCCCAAAGTGCAATGTGCGTTCAA

AAACTGATGATTCACTTCTGCAATTCACAAGAAATATCGCGTTTCGCTGC

GTTCTTCATCGATACGAGAACCAAGAGATCCATTGTTAAAAGTTTTGATT

ATTTTTGTTTTGATTGTATGATTATTGTTTGCTGTGGTAATTTCACAAAT

ATTTTCAATTCTTAATGATCCTTCCGCAGGT

>MW493646(G.C)

TCCTCCGCTTATTGATATGCTTAAGTTCAGCGGGTAATCCTACTTGATCTGAGGTTGAATAGTGTTGTTTTTCAA
ACGAATTTGATTCGATTTTTTAGAAAAGCAATGCAATTCCAAGAGAGAAACAACGCTCAAACAAGTATACTTT
GGGGGATACCCCAAAGTGCAATGTGCGTTCAAAAACTGATGATTCACTTCTGCAATTCACAAGAAATATCGCG
TTTCGCTGCGTTCTTCATCGATACGAGAACCAAGAGATCCATTGTTAAAAGTTTTGATTATTTTTGTTTTGATTG
TATGATTATTGTTTGCTGTGTAAATTTCACAAATATTATCAATTCTTAATGATCCTTCCGCAGGTTCACCTACGG
AAACCTTGTTACGACTTTTACTTCC

>ON844336(ALT.ALT)

TACTGATCCGAGGTCAAAGTTGAAAAAGAGGCTTAATGGAT

GCTAGACCTTTGCTGATAGAGAGTGCGACTTGTGCTGCGCTCCGAAACCA

GTAGGCCGGCTGCCAATTACTTTAAGGCGAGTCTCCAGCAAAGCTAGAGA

CAAGACGCCCAACACCAAGCAAAGCTTGAGGGTACAAATGACGCTTGAAC

AGGCATGCCCTTTGGAATACCAAAGGGCGCAATGTGCGTTCAAAGATTCG

ATGATTCACTGAATTCTGCAATTCACACTACTTATCGCATTTCGCTGCGT

TCTTCATCGATGCCAGAACCAAGAGATCCGTTGTTGAAAGTTGTAATTAT

TAATTTGTTACTGACGCTAATTGCAATTACAAAAGGTTTATGTTTGTCCT

AGTGGTGGGCGAACCCACCAAGGAAACAAGAAGTACGCAAAAGACAAGGG

TGAATAATTCAGCAAGGCTGTAACCCCGAGAGGTTCCAGCCCGCCTTCAT

ATTTGTGTAATGATCCCTCCG

>MZ047482(ALT.ALT)

AGGGGATCTACTGATCCGAGGTCAAAGTTGAAAAAAAGGCTTAATGGATGCTAGACCTTTGCTGATAGAGAG
TGCGACTTGTGCTGCGCTCCGAAACCAGTAGGCCGGCTGCCAATTACTTTAAGGCGAGTCTCCAGCAAAGCTA
GAGACAAGACGCCCAACACCAAGCAAAGCTTGAGGGTACAAATGACGCTCGAACAGGCATGCCCTTTGGAAT
ACCAAAGGGCGCAATGTGCGTTCAAAGATTCGATGATTCACTGAATTCTGCAATTCACACTACTTATCGCATTT

CGCTGCGTTCTTCATCGATGCCAGAACCAAGAGATCCGTTGTTGAAAGTTGTAATTATTAATTTGTTACTGACG
CTGATTGCAATTACAAAAGGTTTATGTTTGTCCTAGTGGTGGGCGAACCCACCAAGGAAACAAGAAGTACGCA
AAAGACAAGGGTGAATAATTCAGCAAGGCTGTAACCCCGAGAGGTTCCAGCCCGCCTTCATATTTGTGTAATG
ATCCCTCCGCAGGTTCACCTACGGAGACCTTGTTACGATTTTTAACTTCCAAA

>ON844337(P.P)

TACCTGATCCGAGGTCACCTGGATAAAAATTTGGGTTGA

TCGGCAAGCGCCGGCCGGGCCTACAGAGCGGGTGACAAAGCCCCATACGC

TCGAGGACCGGACGCGGTGCCGCCGCTGCCTTTCGGGCCCGTCCCCCGGA

ATCGGAGGACGGGGCCCAACACACAAGCCGGGCTTGAGGGCAGCAATGAC

GCTCGGACAGGCATGCCCCCCGGAATACCAGGGGGCGCAATGTGCGTTCA

AAGACTCGATGATTCACTGAATTTGCAATTCACATTACGTATCGCATTTC

GCTGCGTTCTTCATCGATGCCGGAACCAAGAGATCCGTTGTTGAAAGTTT

TAAATAATTTATATTTTCACTCAGACTTCAATCTTCAGACAGAGTTCGGG

GGTGTCTTCGGCGGGCGCGGGCCCGGGGGCGTGAGCCCCCCGGCGGCCAG

TAAAGGCGGGCCCGCCGAAGCAACAAGGTAAAATAAACACGGGTGGGGGA

GGTTGGACCCAAAGGGCCCTCACTCGGTAATGATCCTTCC

>MN856243(P.P)

TTCCTCCCCCTTTTGATATGCTTAAGTTCAGCGGGTATCCCTACCTGATCCGAGGTCAACCTGGATAAAAATTT
GGGTTGATCGGCAAGCGCCGGCCGGGCCTACAGAGCGGGTGACAAAGCCCCATACGCTCGAGGACCGGACG
CGGTGCCGCCGCTGCCTTTCGGGCCCGTCCCCCGGAATCGGAGGACGGGGCCCAACACACAAGCCGGGCTTG
AGGGCAGCAATGACGCTCGGACAGGCATGCCCCCCGGAATACCAGGGGGCGCAATGTGCGTTCAAAGACTC
GATGATTCACTGAATTTGCAATTCACATTACGTATCGCATTTCGCTGCGTTCTTCATCGATGCCGGAACCAAGA
GATCCGTTGTTGAAAGTTTTAAATAATTTATATTTTCACTCAGACTTCAATCTTCAGACAGAGTTCGGGGGTGT
CTTCGGCGGGCGCGGGCCCGGGGGCGTGAGCCCCCCGGCGGCCAGTAAAGGCGGGCCCGCCGAAGCAACA
AGGTAAAATAAACACGGGTGGGAGGTTGGACCCAAAGGGCCCTCACTCGGTAATGATCCTTCCGCAGGCACC
CCTAACGGAAG

>HG326299(P.P)

TTTTTTTTTTTCTTTGCCTTCCCCCCGTTGGTATTATATAGAGGGTGTGCTCCCCCCCCCCTTTGGGGGGAAACA
CCGAAAAAAATTTTGTGTGCGTGCGCCGCGCCGGGGGGGGCTAAAAAGAGAGAGGACAAAACCCCCCCTCC
CGGGGGAGGGACGGGGGTTCGGTGTCTCTTTTGGGCCTCCCCCCCGGAGATAGGAGAGGGGCCCCCCCCAC
ACCACCCGCTGGGCCCAAAAGTCTTGGACAGCCTTGGCCCCCCGGCATGCCCGGGGCCATTGGTTTAAAAGGT
CGGATATTTCACGGAATTGCCAATTTCACCTTTAGGTATTCCATTTGCGTGTTTTTTTAATGCAGCGCAACCCCA
AGGTTTCCTTGTTGGGAGGTTTTTAAATTTTTTTTTTTTTTTCCTTCCGGGCTTCCTTCTTCGGGCCGGTTGGGGG
TTTTTTGGCGGGGCGGGCCGGGGGCTGGGCCCCCGGGCGGCCGGTAAGGCGGCCCGGCGGGGCCCGGGTT
ACCGGGTGGGGGGTGGCCCCCGGGGGGCCTCTGGTACCTTTCGGGTAGGGGTGCTGCGGAAGGATCATTAC
CGAGTGAGGGCCCTTTGGGTCCAACCTCCCACCCGTGTTTATTTTACCTTGTTGCTTCGGCGGGCCCGCCTTTA
CTGGCCGCCGGGGGGCTCACGCCCCCGGGCCCGCGCCCGCCGAAGACACCCCCGAACTCTGTCTGAAGATTG
AAGTCTGAGTGAAAATATAAATTATTTAAAACTTTCAACAACGGATCTCTTGGTTCCGGCATCGATGAAGAAC
GCAGCGAAATGCGATACGTAATGTGAATTGCAAATTCAGTGAATCATCGAGTCTTTGAACGCACATTGCGCCC

CCTGGTATTCCGGGGGGCATGCCTGTCCGAGCGTCATTGCTGCCCTCAAGCCCGGCTTGTGTGTTGGGCCCCG TCCTCCGATTCCGGGGGACGGCCCGAAAGGCAGCGGCGGCACCGCGTCCGGTCCTCGAGCGTATGGGGCT TTGTCACCCGCTCTGTAGGCCCGGCCGGCGCTTGCCGATCAACCCAAATTTTTATCCCAGGTGACCTCGGATCA GGTAGGATACGACAGTAC

>ON844338(F.I)

GGTCACATTCATAAGTTGGGGTTTTAC

GGCGTGGCCGCGACGATTACCAATAACGAGGTGTATGATTACCACACTAG

GAAGCTCGACGTGACCGCCCATCGATTTGGGGAACGCGGGTTACCGCGAG

TCCCAACACCAAGCTGAGCTTGAGGGTTGAAATGACGCTTGAACAGGCTT

GCCCGCCAGAATACTGGCGGGCGCAATGTGCGTTCAAAGATTCGATGATT

CACTGAATTCTGCAATTCACATTACTTATCGCATTTTGCTGCGTTCTTCA

TCGATGCCTAACCCAAGAGATCCGTTGTTGAAAGTTTTGATTTATTTGTT

TGTTTTACTCAGAAGTTCCACTAAAAACAGAGTTTAGGGGTCCTCGGGCG

GGCCGTCCCTTTTTACGGGGCGCGGGCTGATCCGCCGAGGCAACGTATAG

GTATGTTCACAGGGGTTTGGGAGTTGTAAACTCGGTAATGATCCCTCCGC

>MG543801(F.I)

ATATGCTTAAGTTCAGCGGGTATTCCTACCTGATCCGAGGTCACATTCAGAAGTTGGGGTTTTACGGCGTGGC CGCGACGATTACCAGTAACGAGGTGTATGATTACTACGCTATGGAAGCTCGACGTGACCGCCAATCGATTTGG GGAACGCGGGTTACCGCGAGTCCCAACACCAAGCTGAGCTTGAGGGTTGAAATGACGCTCGAACAGGCATGC CCGCCAGAATACTGGCGGGCGCAATGTGCGTTCAAAGATTCGATGATTCACTGAATTCTGCAATTCACATTACT TATCGCATTTTGCTGCGTTCTTCATCGATGCCAGAACCAAGAGATCCGTTGTTGAAAGTTTTGATTTATTTGTTT GTTTTACTCAGAAGTTCCACTAAAAACAGAGTTTAGGGGTCCTCGGGCGGGCCGTCCCTTTTTACGGGGCGCG GGCTGATCCGCCGAGGCAACGTATAGGTATGTTCACAGGGGTTTGGGAGTTGTAAACTCGGTAATGATCCCTC CGCAG

>ON845446(H.V)

GACTTCAAGTCTTGTTGGAGAAATGAAAAGGAATT

TATTGCCTAACGCACGTTAACTATTCCTTGGTCTCTCCCTTCAAGCATAA

TCGTTATACTACCAAGTAAATACAGCGTCTCCAGTAATACCTACTGTTGA

AGGAGAAAAAACAGAAACAGTCTCCAATTTCAAGCTTTTTTTGATTATCG

CCCACGACCGAAAAATAATAAATTATCTTTTGAGAAGGAAATGACGCTCA

AACACGCATGCCCCTGAGAATTTTAAAGGGAGCAATGTGAGTTCAAAAAT

TCAATGATTCACGAGTATCTGCAATTCATATTACTTATCGCAATTCGCTA

CGTTCTTCGGCGATGCGAGAACCAAGAGATCCGTTGTTGAAAGTTTTAAA

TTATTTTAAAATTTCCGTTAGGAATTTTGGTTTAGTTTAAAAAATTATAA

TAAAATAAAATTGTTTGTGTTTGTTTTTTGCCTTGAACCTTTCGATTCAA

AGC

>MK613264(H.V)

ACGCCGAACCAGCCATGATTTGAGGTCACACTTGATGAATATTAAAAGCAACCCTTTGCCTAAGGTACGTTAC
CATTTCCCTTGTAAAGTAAAACGAATAAATCCATAAATACATCACAGCGAGAACAGCGTCTCCAAAGAAGCTA
AGTGTTGAATTAAAAAAGACTGAAACAGTCTCCAATTTCAAGCTAACCCTGAGTATCGCCCACAACCAAAAAA
TAATAAATTATCTTTTGAGAAGGAAATGACGCTCAAACAGGCATGCCCCTGAGAATGCTCAAGGGCGCAATGT
GCGTTCAAAAATTCAATGATTCACGAGTATCTGCAATTCACATTACTTATCGCAATTCGCTACGTTCTTCATCGA
TGCGAGAACCAAGAGATCCGTTGTTGAAAGTTTTAAATTATTTTAAAATTTCCGTTAGGAATTTTGGTTTAGTT
TAAAAAATTATAATAAAATAAAATTGTTTGTGTTTGTTTTTTGCCTTGAACCTTTCGATTCAAAGCAGAAAGAAT
TAAATTAAAGTAAAAAACTCCAATGTGTGTAGCGTTGACTGAGATTCAAGCAAGACTACTTTCACTGCGACAC
TCTTAAGAAGCAGCGCAATTAAGCGCACATCTTCAATAAAGATACACATTATTGTAAAAGATCTAAACAAGAA
CTCGAGCAACAATGATTATTCAATCTAATGATCCTTCCGCAGGTACCCCTA

>BHU118(Ascomycota.sp)

TGATCCGAGGTCACCTGGATGAAAGTTTGGTGTGATA

TCGCCGGCGCCCGGCAGGCCTACCGAACGGGGGGAAAACCCCCAACCCCT

GAAGAACGGAAGCCGTGCCCCCCCTTCCCTTTCGGGCCCTCCCCCCGGAA

AGAAGAAAGAACCCAACCCACAAACCCTGCTTGAAGGGGCCATGACCCTT

GGAAAGGGGCCCCCCCGAATACCCCAAGGGGCAATGGGCGTTCAAAGACT

CCATGAATCACTGAATTTTGCAATTTCATTACGTAACGCAATTTCCTGCG

TTCTTCCACCATTCCCGAACCCAAAAATCCCTTTTTTAAAGTTTTAAATA

ATTTAAATTTTGTCTCAAAACAACATC

>OK095335(A.F)

CTTAGAAAAATAAAGTTGGGTGTCGGCTGGCGCCGGCCGGGCCTACAGAGCAGGTGACAAAGCCCCATACGC
TCGAGGACCGGACGCGGTGCCGCCGCTGCCTTTCGGGCCCGTCCCCCGGGAGAGGGGGACGGGGGCCCAAC
ACACAAGCCGTGCTTGAGGGCAGCAATGACGCTCGGACAGGCATGCCCCCCGGAATACCAGGGGGCGCAAT
GTGCGTTCAAAGACTCGATGATTCACTGAATTCTGCAATTCACATTACTTATCGCATTTCGCTGCGTTCTTCATC
GATGCCGGAACCAAGAGATCCGTTGTTGAAAGTTTTAACTGATTACGATAATCAACTCAGACTGCATACTTTCA
GAACAGCGTTCATGTTGGGGTCTTCGGCGGGCGCGGGCCCGGGGGCGCAAGGCCTCCCCGGCGGCCGTCGA
AACGGCGGGCCCGCCGAAGCAACAAGGTACGATAGACACGGGTGGGAGGTTGGACCCAGAGGGCCCTCACT

CGGTAATGATCCTTCCGCAGGTCCACTTACGGAAGATCAACCGANGTGAGGGCCCTCTGGGTCCACCTCCACC
CGTGTCTTCTACTTGTT

>MW857110(P.B)

GTCAGCCTGGAAGAAGTTTGGTATGATCGGCAGGCGCCGGCCAGTCCTACAAAGCGGGTGACAAAGCCCCAT
ACGCTTGAGGACCGGACGCGGTGCCGCCGCTGCCTTTCGGGCCCGTCGACCGGAAGGAGGACAGAGGGGAA
CAGAAAGCCGAGCTTGAGGGCATCAATGACGCTCGGACAGGCATGCCCTCCGGAATACCAGAGGGCGCAAT
GTGCGTTCAAAGACTCGATGATTCACTGAATTCTGCAATTCACATTACGTATCGCATTTCGCTGCGTTCTTCATC
GATGCCGGAACCAAGAGATCCGTTGTTGAAAGTTTTAAATAATTTATATTTAATCTCAGACTACAATCTTCAGA
CAGAGTTCTAAGGTGTCTTCGGCGAGCGCGGACCCGGGGACAGACGTCCCCCGGCAGCCAAAAGGCAGGCT
CGCCGAAGCAACAAGGTAAAATAAACACGGGTGGGAGGTTGGACCCAGAGGGCCCTCACTCGGTAATGATC
CTTCCGCAGGTTCACCCTACGGGAGGATCATTACCGAGTGAGGGCCCTCTGGGTCCACCTCCACCGTGTTTATT
TTACCTTGTTGCTTCGGCACCTGCCTTTTGGCTGCCGGGGGGACGTCTGTCCCGGGTCGCGCTCCGAAAA
